# Using Complete Genome Comparisons to Identify Sequences Whose Presence Accurately Predicts Clinically Important Phenotypes

**DOI:** 10.1371/journal.pone.0068901

**Published:** 2013-07-23

**Authors:** Barry G. Hall, Heliodoro Cardenas, Miriam Barlow

**Affiliations:** 1 Bellingham Research Institute, Bellingham, Washington, United States of America; 2 University of California Merced, Merced, California, United States of America; Oregon State University, United States of America

## Abstract

In clinical settings it is often important to know not just the identity of a microorganism, but also the danger posed by that particular strain. For instance, *Escherichia coli* can range from being a harmless commensal to being a very dangerous enterohemorrhagic (EHEC) strain. Determining pathogenic phenotypes can be both time consuming and expensive. Here we propose a simple, rapid**,** and inexpensive method of predicting pathogenic phenotypes on the basis of the presence or absence of short homologous DNA segments in an isolate. Our method compares completely sequenced genomes without the necessity of genome alignments in order to identify the presence or absence of the segments to produce an automatic alignment of the binary string that describes each genome. Analysis of the segment alignment allows identification of those segments whose presence strongly predicts a phenotype. Clinical application of the method requires nothing more that PCR amplification of each of the set of predictive segments. Here we apply the method to identifying EHEC strains of *E. coli* and to distinguishing *E. coli* from *Shigella*. We show *in silico* that with as few as 8 predictive sequences, if even three of those predictive sequences are amplified the probability of being EHEC or *Shigella* is >0.99. The method is thus very robust to the occasional amplification failure for spurious reasons. Experimentally, we apply the method to screening a set of 98 isolates to distinguishing *E. coli* from *Shigella*, and EHEC from non-EHEC *E. coli* strains and show that all isolates are correctly identified.

## Introduction

Clinically important bacterial phenotypes can be difficult, expensive and time-consuming to determine. Phenotypic assays require expensive reagents and growth of the bacteria. PCR assays which detect genotype do not always correctly indicate phenotype. However, genotype and phenotype in organisms as simple as bacteria should have strong correlations, and whatever disconnect exists between genotype and phenotype likely results from an incomplete capture of genotype in which too few genetic features are considered.

Most bacterial species are characterized by a “pan-genome” in which there is a set of genes that are present in all members of the species (core genes) and a large set of genes each of which is present in some, but not all, members of the species (accessory genes) [1]–[4]. The major fraction of variation among bacterial genomes of the same species derives not from base substitutions, but from massive rearrangements coupled with massive gain/loss of large DNA segments. The fraction of the genome that is accessory genes ranges from ∼8% (*B. anthracis, M. tuberculosis*) to ∼35% (*E. coli*) (Hall, unpublished results based on analysis of 22 species using methods described in [1]).

That variance in genetic content makes bacterial genomes particularly difficult to compare because genome comparison requires comparing homologous DNA sequences. To ensure comparisons among homologous bases, genes are typically aligned by one of a variety of multiple sequence alignment (MSA) methods that introduce into the alignment gaps that are intended to represent historical insertions or deletions (indels). That approach reflects the assumptions that individual genes evolve primarily by base substitutions and indels, and it works well for sequences which meet those assumptions. MSA is not robust when those assumptions are violated. MSA methods generally fail when inversions, transpositions and many indels occur.

To overcome those difficulties in bacterial genome alignment, we have developed and applied a novel approach which we have named the “Bop” method [5]. The Bop method produces a description of each genome as a binary string that indicates the presence or absence of each bop that is to be found among the strains that are analyzed. Because bops are short homologous sequences, the set of binary strings constitutes an automatic alignment of the set of genomes with respect to the presence/absence of those bops. The bop alignment can be used directly to estimate relationships among the strains.

The most common way to estimate the relationships among organisms is by phylogenetic analysis, but phylogenetic analysis is not always appropriate for the set of organisms being compared or for the data that is used to characterize those organisms. Phylogenetic trees are used to estimate the relationships of organisms to hypothetical ancestors, and thereby to each other. The branches on a phylogenetic tree are intended to reflect the number of mutations that separate an organism from its hypothetical ancestor, or one of those ancestors from its immediate ancestor. There are two fundamental assumptions involved in phylogenetic analysis, neither of which applies to these data sets. First, characters that are shared between a pair of individuals are assumed to be identical by descent. Deviations from that assumption, collectively called *homoplasies*, may arise from convergence, incomplete genetic isolation, etc and are indications of loss of phylogenetic signal. For these data there is no implication that shared characters are identical by descent. A pair of strains that have the same bop may do so because they inherited that bop from a common ancestor, because of exchange with a distantly related individual, or because each has acquired the same plasmid or phage-borne bop. Similarly, individuals that lack a particular bop may do so because of inheritance or because each has independently suffered loss of that bop. The nature of the data makes the use of phylogenetic analysis inappropriate. Second, phylogenetic analysis assumes that the individuals are genetically isolated from each other; i.e. they do not exchange genetic information and inheritance is strictly vertical. As a result a node, internal (ancestral node) or external (extant individual), can have only one ancestor. That assumption certainly does not apply to most microbial species where genetic exchange among individuals is common, and in particular does not apply to *E. coli* which is known to undergo considerable genetic exchange [6]. These considerations indicate that phylogenetic analysis is not appropriate for estimating the relationships among these genomes.

A more appropriate approach to this problem is a *spanning tree*, which is a subset of a fully connected graph in which there is a single path from any node to any other node. A *minimum spanning tree* (MST) is the shortest spanning tree of all the possible spanning trees. Depending on the order in which the nodes are considered it is possible for there to be more than one MST [7]. MSTs are widely used in microbial epidemiology to represent relationships among strains. MSTs make no assumptions about identity by descent or the absence of genetic exchange. MSTs are based only on identity by state, thus the relationships that are diagramed only indicate the overall similarities among the individuals.

To obtain an MST, the bops are combined into segments, where a segment is a contiguous series of bops that have identical distributions among the set of genomes, and a new binary string is written that describes the presence/absence of each segment in the genome.

The segment alignment can then be used to determine how tightly each segment is associated with a phenotype of interest. Segments that are always present (or always absent) in strains with the phenotype of interest, and are always the opposite when the phenotype is not expressed are candidates for amplification by PCR to estimate the probability that an unknown strain exhibits that phenotype.

Given the abundance of complete genome sequence data for numerous strains expressing the same phenotype, we thought it might be possible to identify multiple genetic markers and to establish the probabilities of certain phenotypes being expressed based on the presence or absence of those genetic markers. We assumed that many clinically important phenotypes are determined less by variation within homologous sequences (SNPs) than by the presence or absence of accessory genes within the genome. Identification of accessory sequences associated with pathogenic phenotypes requires comparison of genomic sequence obtained from strains whose phenotypes are known.

We used complete genome sequences in order to identify sequences whose presence is strongly associated with difficult-to-determine phenotypes. Once identified, we reasoned that amplification of such sequences would provide a rapid, reliable and inexpensive means of assessing the probabilities of those phenotypes. We have applied this method to two clinically important species of bacteria, *Escherichia coli* and *Shigella.*

*Escherichia coli* K12 was among the first bacteria to be completely sequenced [8]. Its historic role as the laboratory strain that was the center of the development of molecular biology fully justified sequencing its genome. Long regarded as a benign commensal, that perspective changed in 1982 when an O157:H7 enterohemorrhagic (EHEC) strain was shown to be responsible for the “Jack-in-the-box” outbreak that resulted in several deaths. Sequencing an O157:H7 strain [9] surprised the microbial community by revealing the dramatic differences in gene content and gene arrangement between the two sequenced genomes. *E. coli* is now well understood to vary enormously with respect to pathogenicity, and a variety of pathogenic phenotypes have been described including enterotoxigenic (ETEC), adherent-invasive (AIEC), enteroaggregative (EAEC), enteropathogenic (EPEC), and extraintestinal pathogenic (ExPec). Other strains are recognized as non-pathogenic commensals, of which five (K12 and its derivatives, B and its derivatives, C, W, and “Crookes”) are considered “safe” strains for general laboratory use and are classified as Risk Group 1[10]. As of November 2011 46 *E. coli* strains have been completely sequenced.

Enterohaemorrhagic *E. coli* (EHEC) causes serious symptoms including lower gastrointestinal bleeding, diarrhea and colitis. The clinical importance of these strains, and the need to track outbreaks and epidemics of these pathogens means that properly identifying them is important. However current clinical assays often fail to tell them apart because they are closely related, and symptomatically similar. Because *E. coli* varies so much in pathogenicity it is often not sufficient for public health officials to simply determine whether or not *E. coli* is present, it is often important to determine the danger posed by the strains that are present. Serotyping is an important tool in evaluating risk (O157:H7 strains can be presumed to be EHEC and therefore very dangerous), but other serotypes are also EHEC and it is not necessarily the case that all members of a particular serogroup would be EHEC. Determination of the EHEC phenotype is neither rapid nor cheap. O serotyping is performed following the procedure published by Orskov et al. [11]. H typing is performed by the method described by Machado et al. [12].

It would be useful to identify DNA sequences that correlate very strongly with the EHEC phenotype in order to develop a PCR assay that could quickly determine the probability that a given strain is EHEC. It is well understood that many of the virulence determinants associated with the EHEC phenotype are plasmid borne, but although some plasmids are shared by EHEC strains, none is shared by all.

*Shigella* is another serious pathogen that infects the digestive tract and can cause abdominal cramping, lower gastrointestinal bleeding, diarrhea, and colitis and severe dehydration. *Shigella* has been treated as a separate genus because of its clinical pathogenicity resulting in shigellosis, but it has long been considered to be part of *E. coli* [13], [14]. At this time eight *Shigella* strains have been completely sequenced. *Shigella* is a clinically important clade within *Escherichia coli* [15] that causes shigellosis [16]. Distinguishing *Shigella* from other *E. coli* is non-trivial, and it would be valuable to have molecular markers that would unambiguously distinguish the two.

Here we apply the Bop method to the analysis of 47 *E. coli* and 8 *Shigella* completely sequenced genomes to determine their similarity and to identify genetic sequences that correlate strongly with the phenotype of species identity for EHEC *E. coli* and *Shigella*, with the intent of creating a reliable PCR assay for rapidly identifying them.

## Results

### BopGenomes analysis of E. coli –Shigella genomes

The 47 *E. coli* genomes and 8 *Shigella* genomes were analyzed using ***BopGenomes***. table S1 lists the GenBank accession numbers, pathogenicity phenotypes and references for those phenotypes. The 55 genomes were analyzed both including and excluding plasmids. In each case the genomes were digested *in silico* by restriction enzyme *NcoI*. When plasmids were excluded, the 55 genomes included 17,469 unique restriction fragments and comprised 69,010 unique bops. When plasmids were included, there were 18,193 unique restriction fragments and 76,517 bops.

### Clustering of *E. coli-Shigella* genomes based on bops

**Figure 1:**
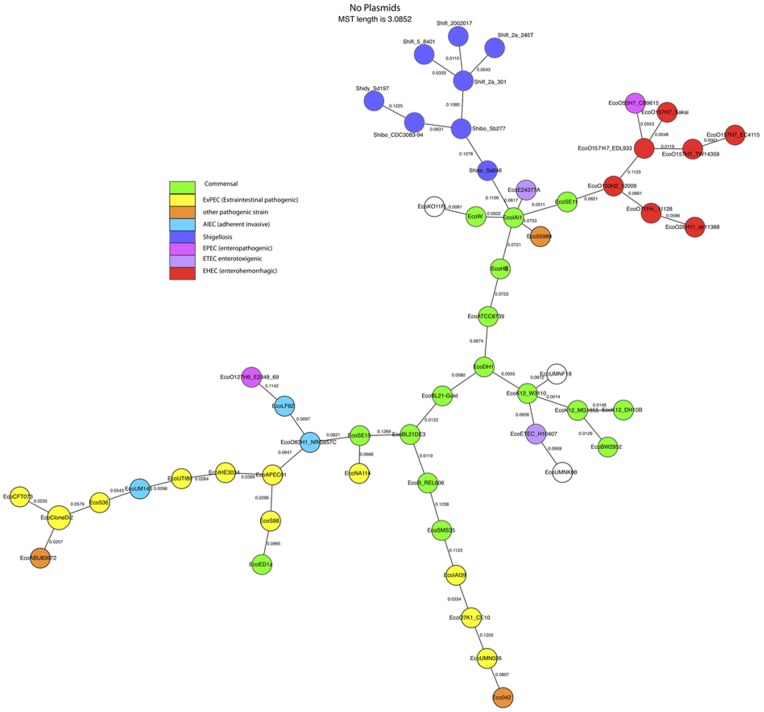
Minimum spanning tree based on complete genomes excluding plasmids. Pathogenicity phenotypes are indicated by colors. The pathogenicity of uncolored strains is not known. Full strain IDs and accession numbers of the genome sequence files are given in Table S1.

**Figure 2:**
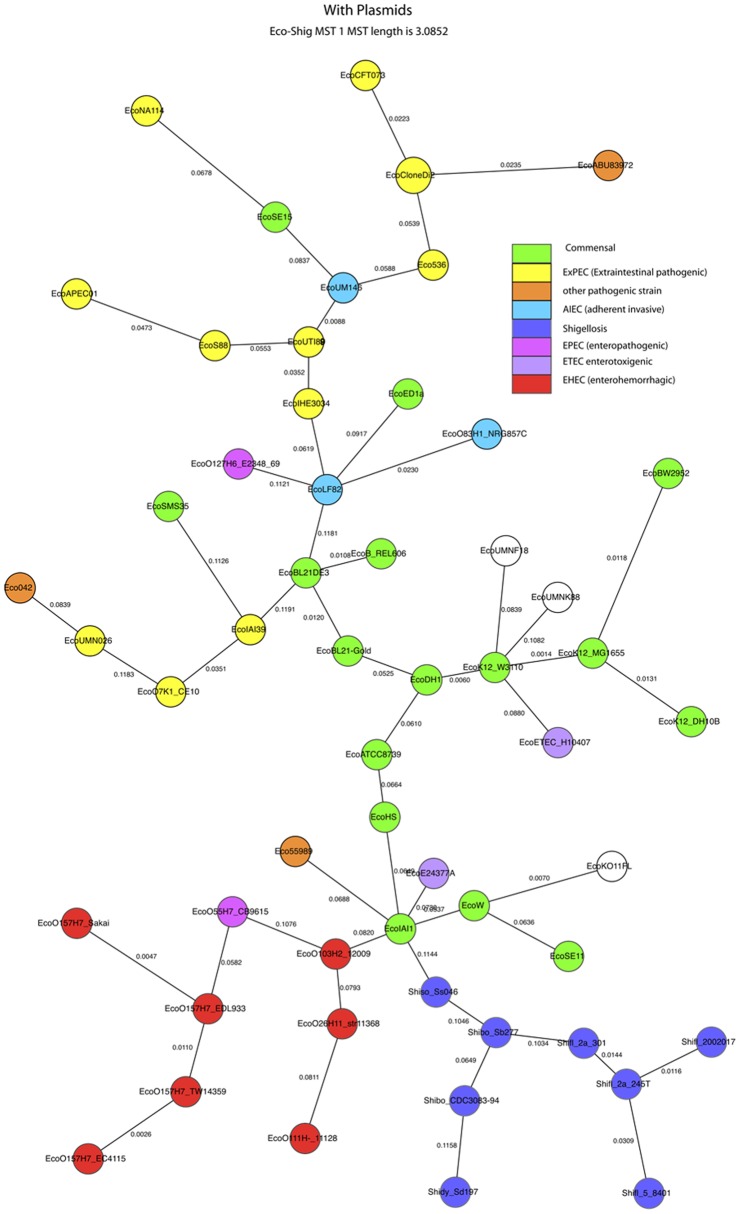
Minimum spanning tree based on complete genomes including plasmids. Pathogenicity phenotypes are indicated by colors. The pathogenicity of uncolored strains is not known. Full strain IDs and accession numbers of the genome sequence files are given in Table S1.

Minimum spanning trees (MST) based on the presence or absence of each bop are shown in Figures 1 and 2. For the analysis shown in Figure 1, we excluded plasmids. For Figure 2, plasmids were included.

Figures 1 and 2 have been colored to indicate the pathogenic phenotypes when those are known. In general phenotypes tend to cluster together. We define a cluster as a set of genomes such that there is a path from each member of a cluster to every other member of that cluster, and the path does not pass through any node not belonging to that cluster. When plasmids are excluded the EHEC strains fall into a single cluster, whereas when plasmids are included they fall into two closely related clusters, one consisting of the four O157:H7 strains. In both data sets the *Shigella* strains fall into a single cluster, and 13 or 14 of the 16 the commensal strains fall into a single cluster.

In both of these cases there was a single MST. The two MSTs do differ in some details. When plasmids are excluded (Figure 1) the three AIEC strains form a cluster; when plasmids are included (Figure 2) they do not. One of those strains (LF82) lacks plasmids, each of the others has a large (>100 kb) plasmid but the plasmids are unrelated to each other.

### Predicting phenotypes and identifying sequences that do so

We asked whether there are any chromosomal DNA sequences that are common to, and perhaps exclusive to, EHEC strains. To answer this question we used ***BopGenomes*** to identify EHEC-specific segments. in a sequence data set (See Materials and Methods) in which plasmids were excluded.

**Figure 3:**
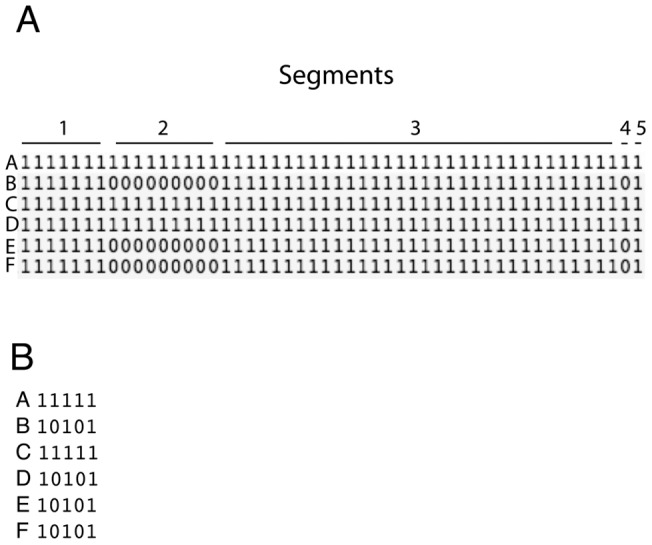
Determination of segments from bops. A: Part of a .scores file in which binary strings indicate the presence or absence of a series of bops for strains A–E. Numbered lines above the string show contiguous bops that are identically distributed among strains A–E. B: A corresponding .segScores file in which the binary strings indicate the presence of absence of the segments shown in panel A.

Each segment consists of a contiguous series of bops that have identical distributions among the set of genomes. ***BopGenomes*** generates a file (.segScores) in which each strain is described by a binary string that shows the presence/absence of each segment (Methods and Figure 3). The program ***GetProbs*** was used to determine, for each segment, the probability that it is present in a set of EHEC strain and in a set of non-EHEC strains. That set of strains is known as the “training set” and consisted of four randomly chosen EHEC strains and 30 randomly chosen non-EHEC strains. A parameter, ß, was calculated for each segment. ß is the probability that the presence/absence of the segment is non-randomly distributed with respect to the phenotype. The output of ***GetProbs*** was used by the program ***PredictPhenotypes*** (1) to calculate for all 55 genomes the probability that the strain is EHEC and (2) to identify the sequences that most strongly predict the EHEC phenotype.

Of the 47 *E. coli* strains 7 are known to be EHEC and 38 are known to be non-EHEC (Table S1). To test the predictive ability of our programs 4 EHEC strains and 30 non-EHEC strains (including *Shigella* strains) were used as training strains to predict the EHEC phenotypes of the remaining strains (of which 3 were known to be EHEC and 16 to be non-EHEC). Given the small number of EHEC strains the predictions are expected to vary slightly depending upon the makeup of the training set. With that in mind 20 independent runs of ***GetProbs*** followed by ***PredictPhenotypes*** were carried out. In each run only sequences with a ß value >0.9999999 were used to predict phenotypes and to identify predictive sequences. Twenty runs provide a reasonable fraction of the possible combinations of 4 EHEC and 30 non-EHEC strains in the training sets.

Table 1 shows the probabilities with which each EHEC strain was predicted to be EHEC over the 20 runs. Among the non-EHEC strains the mean probability of being EHEC was 0.0098, with the maximum probability being 0.19 for strain UMNK88. Together with the results in Table 1 this provides an excellent example of the ability of this analysis to predict phenotypes based on the presence of segments with high ß values.

**Table 1:** EHEC strains.

Strain	Probability of being EHEC
EcoO157H7_EC4115	1.00
EcoO157H7_EDL933	0.99
EcoO157H7_Sakai	0.99
EcoO157H7_TW14359	1.00
EcoO26H11_str11368	0.84
EcoO103H2_12009	0.85
EcoO111H-_11128	0.88

Eighteen segments had ß values >0.9999999. A better test of the predictive utility of those 18 segments comes from blast searches of the non-redundant (NR) nucleotide database. Table 2 shows the results of those searches. Nearly all of the segments (except 13 and 16) show homology with more EHEC than non-EHEC hits. Eight of the segments would be useful as PCR probes. The sequences of those eight segments are given in table S2. While most of those eight segments on their own would have insufficient predictive ability for reliable clinical assays, when combined, they are powerful predictors of phenotype. Based on the *in silico* specificities, any strain in which even the four least specific probes amplified would have a >99.97% probability of being an EHEC *E. coli*.

**Table 2:** Segments used to search the nr database.

Segment	Segment ID	Length (bp)	Number of EHEC hits^a^	Number of non-EHEC hits^a^	p^b^	Comment^e^
1	10254	734	7	0	1.0	+
2	10258	6,924	7	1^c^	0.875	+
3	10261	200	7	2^c^	0.778	
4	10263	270	7	1^c^	0.875	+
5	10314	600	7	1	0.875	+
6	10375	108	7	1^c^	0.875	
7	10380	196	7	2	0.778	
8	10391	217	7	2^c^	0.778	
9	10393	400	7	0	1.0	+
10	10396	800	7	0^d^	1.0	+
11	10398	141	7	1^d^	0.875	
12	10402	636	7	0	1.0	+
13	10545	12,387	7	12	0.368	
14	10547	1,744	7	3	0.700	
15	10549	376	7	1	0.875	+
16	10551	5,590	7	14	0.333	
17	10605	134	7	0	1.0	
18	11916	138	7	1^c^	0.875	

a Hits align over at least 50% of the query length.

b p is the probability that a hit is EHEC.

c Includes *Citrobacter rodentium* ICC168, a strain that is known to have acquired EHEC and EPEC associated sequences from *E. coli* [21].

d Hits in bacteriophage genomes were not counted.

e Plus sign indicates that length is ≥200 bp and p is >0.80. Segments indicated by + would be useful as PCR probes to detect EHEC strains.

### Identification of Shigella-specific sequences

We used ***GetProbs*** to identify 8 sequences, ranging from 400 to 1536 bp, that were present only in the 8 *Shigella* strains (ß≥0.9999999999). We used each of those sequences as queries in BLAST searches to screen the entire non-redundant database of DNA sequences. None of the sequences were present in any organism but *Shigella*, including in any of the 47 completely sequenced *Escherichia coli* strains. Our criterion for being present was that the query aligned over >50% of its length with >80% sequence identity. All but one of the sequences was present in all eight of the completely sequenced *Shigella* strains and none were present in any other organisms including *E. coli*. Some sequences did align over short regions with other organisms, so we trimmed the query sequences to include only the completely *Shigella*-specific regions (Table S3).

### Experimentally testing the reliability of EHEC-specific PCR amplification probes

We identified primers for each of the EHEC-specific sequences in Table S2 (Table S4) and screened a collection that included 56 EHEC *E. coli*, 17 non-EHEC *E. coli*, 16 *Shigella sp.*, 4 *Klebsiella pneumoniae*, 1 *Klebsiella oxytoca*, 3 *Proteus mirabilis*, and 1 *Pseudomonas aeruginosa* strains. Three of the EHEC strains and two of the non-EHEC strains were among the set for which complete genome sequences are available. Genomic DNA was prepared from each strain and was used as the template for PCR reactions with each pair of primers. Four of the probes, EHEC 2, EHEC3, EHEC 7 and EHEC8, were deemed unreliable on two grounds: (a) they produced amplicons in less than half of the known EHEC strains, and (b) they failed to produce amplicons in the three EHEC strains that had been completely sequenced an in which the sequences were known to be present (Table 3). None of the four reliable EHEC probes amplified all 56 EHEC strains, and one probe, EHEC 4, amplified two *Shigella* strains. Thus none of the probes is, by itself, capable of reliably identifying EHEC strains.

**Table 3:** Experimental reliability of EHEC-specific and Shigella-specific PCR probes.

EHEC-specific PCR probes		
Probe	Number of amplicons	Number of amplicons not in EHEC strain
EHEC 1	48	0
EHEC 2	27	0
EHEC 3	0	0
EHEC 4	44	2
EHEC 5	49	0
EHEC 6	47	0
EHEC 7	0	0
EHEC 8	0	0

*Shigella*-specific PCR probes		
Probe	Number of amplicons	Number of amplicons not in *Shigella* strain
Shi 1	15	0
Shi 2	14	0
Shi 3	13	0
Shi 4	17	2
Shi 5	16	0
Shi 6	14	0
Shi 7	14	0
Shi 8	16	0

### Experimentally testing the reliability of *Shigella*-specific PCR amplification probes

We identified primers for each of the 8 trimmed *Shigella*-specific sequences in Table S3 (Table S5), and screened the same collection of bacterial strains. None of the PCR probes produced amplicons in any of the species other than *Shigella* and *E. coli*. Table 3 summarizes the results of those experiments. Only one probe amplified all 17 *Shigella* strains, and one probe, Shi 4, amplified two *E. coli* strains. Again, none of the probes is, by itself, capable of reliably identifying *Shigella*.

### Updating the *in silico* results

In the time since the EHEC-specific and *Shigella*-specific sequences were identified, while the experimental PCR studies were being conducted, an additional nine *E. coli* and two *Shigella* genomes have been completed and one *E. coli* genome has been delisted by GenBank. Minimum spanning trees that have been updated to reflect those changes are shown in figures S1 and S2. The Shigella-specific and EHEC-specific probes (Tables S4 and S5) were used as queries in BLAST searches of the non-redundant nucleotide database. Table 4 shows that both the *Shigella*-specific and EHEC-specific probes are highly specific, but are individually insufficient to identify isolates as EHEC or as *Shigella* with ≥99.9% confidence.

**Table 4:** *In silico* reliability of EHEC-specific and Shigella-specific PCR probes.

EHEC-specific PCR probes		
Probe	Number of hits^a^	Number of hits not in EHEC strain
EHEC 1	10	1
EHEC 2	10	1
EHEC 3	9	0
EHEC 4	9	0
EHEC 5	9	0
EHEC 6	9	0
EHEC 7	10	1
EHEC 8	10	1

*Shigella*-specific PCR probes		
Probe	Number of hits	Number of hits not in *Shigella* strain
Shi 1	11	1
Shi 2	11	1
Shi 3	11	1
Shi 4	11	1
Shi 5	12	2
Shi 6	11	1
Shi 7	11	1
Shi 8	10	1

a Hits in a BLAST search of the GenBank non-redundant nucleotide database.

### Predicting EHEC or Shigellosis phenotypes based on amplification profiles

Practical application of this approach to predicting phenotypes means predicting a phenotype from the ‘amplicon profile’ of a strain. The amplicon profile is a binary string in which a 1 means that a PCR probe produced an amplicon and a 0 means that it did not. For example, *Shigella flexneri* 2a str. 2457T produces BLAST hits (*in silico* equivalent of an amplicon) with all *Shigella-*specific probes except probe Shi 8. Its amplicon profile is therefore 11111110. Table 5A combines the results from Tables 3 & 4 to show, for each probe, the fraction of amplicons or hits that were from non-*Shigella* or non-EHEC strains. Table 5A is based upon a total of 26 *Shigella*, 63 EHEC-*E. coli*, 65 non-EHEC *E. coli*, and 9 strains of other species.

**Table 5:** Probe reliabilities.^a^

EHEC probe	Probability not EHEC^b^	*Shigella* probe	Probability not *Shigella* c
EHEC 1	0.018	Shi 1	0.039
EHEC 2	Unreliable	Shi 2	0.04
EHEC 3	Unreliable	Shi 3	0.042
EHEC 4	0.039	Shi 4	0.107
EHEC 5	0	Shi 5	0.037
EHEC 6	0	Shi 6	0.04
EHEC 7	Unreliable	Shi 7	0.04
EHEC 8	Unreliable	Shi 8	0.039

a Combined results from tables 3 and 4.

b Probability that a strain with a hit or amplicon from this probe is not EHEC.

c Probability that a strain with a hit or amplicon from this probe is not *Shigella.*

While the presence or absence of an *in silico* hit can be considered a completely reliable indicator of the presence or absence of a query sequence in the genome, the presence or absence of an amplicon in a PCR experiment is a much less reliable indicator of the presence or absence of a sequence. The absence of an amplicon can either mean that the sequence is not present in that strain, or that the PCR reaction failed for spurious reasons; i.e. a false negative. False positives are much rarer that false negatives because to be counted as a positive not only must an amplicon be present, it must be an amplicon of the correct size. In other words, we can trust the interpretation of the presence of an amplicon more than we can the interpretation of the absence of an amplicon.

If the amplification profile of an isolate that was probed with all eight *Shigella*-specific probes is 00011001 we want to know the probability that an isolate in which probes Shi 4, Shi 5 and Shi 8 produce amplicons is *Shigella*. Probe Shi 4 produced *in silico* hits in 11 strains Table 4) and amplicons in 17 strains (Table 3), of which a total of 3 strains were not *Shigella*. The probability that a Shi 4 hit or amplicon was not in a *Shigella* strain is therefore 0.107 (Table 5). The probability that a strain with the amplification profile 00011001 is **not** *Shigella* is the product of those probabilities for probes Shi 4, Shi 5 and Shi 8, or 1.54×10^−4^. The probability that the strain is *Shigell*a is therefore 0.9998.

Most *Shigella* strains produced amplicons/hits with all eight *Shigella*-specific probes, and most non-*Shigella* strains produced no hits with any *Shigella*-specific probes, i.e. the amplicon profiles were 11111111 and 00000000 respectively. Their respective probabilities of being *Shigella* are 0.99999999998 and 1.6×10^−11^ respectively. Similarly most EHEC *E. coli* had amplicon profiles of 1111 with the reliable EHEC-specific probes, while most non-EHEC *E. coli* and other species had a 0000 profile. Their respective probabilities of being EHEC are 0.99999993 and 7×10^−8^ respectively. Those strains with other profiles, and their probabilities of being EHEC or *Shigella*, are shown in Table S6.

All of the *Shigella* strains tested were identified as *Shigella* with probabilities ≥0.999. One *E. coli* strain, strain 53638, was incorrectly identified as being *Shigella* with a probability >0.9999, giving a false positive frequency of 1 out of 75 *E. coli* screened, or 0.013 (Table S6). Two EHEC strains, RDEC-1 and RD8, were not identified as EHEC, giving a false negative rate of 0.03.

For clinical applications we suggest that any strain in which the probability of being EHEC or *Shigella* is <0.999 should be rejected as having that phenotype. Because false positives do occasionally occur we suggest that any strain in which a single probe produces an amplicon should be retested.

## Discussion

*Shigella* and *E. coli* have classically been distinguished on the basis of a variety of biochemical and serological tests. More recently they have been distinguished on the basis of presence of the pINV plasmid and of the Shiga toxin genes, but none of these phenotypes are unique to *Shigella*, and in particular many are shared by EIEC *E. coli* strains that closely resemble *Shigella* [17], [18]. Similarly, EHEC *E. coli* are classically distinguished from non-EHEC *E. coli* on the basis of serotype and the presence of Shiga toxin (verocytotoxin), but again those factors are not unique to EHEC strain. The most common EHEC serotype is O157:H7, but there are other EHEC serotypes [15]. Non-EHEC strains, including EIEC and EAEC-STEC strains produce the Shiga toxin [19]. Indeed, “EHEC are sometimes difficult to identify” and “There is no single technique that can be used to isolate all EHEC serotypes” [20]. Because the infectious doses of both *Shigella* and ETEC-*E. coli* are about four orders of magnitude lower that that of most other pathogenic *E. coli* [17], rapid and reliable identification of those organisms is clinically important. The major advantage of using the Bop method to identify phenotype-specific sequences that can be used as PCR probes is not only specificity, but also that using sets of those probes allows a probability statement about the reliability of identification of EHEC and *Shigella* strains to be made. Additionally, use of such probe sets is both rapid and inexpensive.

At present it is quite difficult to obtain phenotypic information about pathogens that have been completely sequenced. It is typically the case that the group that sequenced the strain is interested in only one phenotype (if any). For instance, the public sequence databases rarely include any information about antibiotic resistance. The availability of the Bop method makes a strong argument for developing collections of clinically important strains whose complete genome sequences have been determined and whose phenotypes are thoroughly described. The availability of such a collection would make it possible to develop databases of their phenotypes and to use those phenotypes to identify phenotype-specific PCR probes. Appearance of a new clinically relevant phenotype could quickly be followed by characterization of that collection by a laboratory with the necessary expertise, and in turn followed by development of phenotype-specific PCR probes.

The Bop method can be applied to any species for which a sufficient number of strains in which the phenotype of interest is known have been completely sequenced. The method is useful for estimating the relationships among the sequenced strains, but the most valuable application of the method is to identify phenotype-specific sequences that can then be used to inexpensively and quickly characterize clinical isolates by PCR. Once the bop and segment profiles of a set of sequenced genomes has been determined it requires only a few minutes to identify phenotype-specific segments among those isolates.

Depending upon the intended downstream application of the analysis, one might include the plasmids in each strain or exclude them from the analysis. Excluding plasmids results in the loss of information, and it seems reasonable that two strains that share a plasmid are more alike than they would be did they not share that plasmid. On the other hand, plasmids clearly move more frequently among strains than do chromosomal genes and if one's interest is more in chromosomal similarity, then excluding plasmids makes sense.

These approaches that we have introduced have the potential to apply genomic data for epidemiological and clinical analyses. We believe that the approach of using bops as input for MSTs and segments as a method for identifying sequences that predict phenotype can ultimately be implemented into clinical labs as cost and time effective methods for analyzing infective bacteria. Additionally, the programs we have developed are readily available and can be implemented by a wide range of users with common computing equipment. Our results are also of theoretical importance because they identify a new type of character, namely a bop, to perform robust analyses of genomic sequence data.

## Materials and Methods

### The Bop method

The Bop method is applied to a set of completely sequenced (closed) genomes through use of the ***BopGenomes*** program. The genomes are digested *in silico* with one of several restriction enzymes to produce an *ordered* restriction map. The selection of restriction enzymes is designed to allow adequate restriction of any genome regardless of GC content, and other sequence biases. By allowing the option to digest the genome at a variety of restriction sites, it is possible for the program to accommodate most genomes. Each fragment is given an ID that consists of its length (rounded to the nearest 100 bp) and the lengths of the fragments that flank it. Thus a 16,542 bp fragment flanked by a 4,320 bp to its left and a 1,680 fragment to its right would be identified as 4.3-16.5-1.7. That particular 16.5 kb fragment is distinguished from all other 16.5 kb fragments by the lengths of its flanking fragments. At this point that system appears sufficient to uniquely identify restriction fragments. Cases of multiple occurrences of the same fragment turn out to be duplicated regions that contain the same internal restriction fragments. Usually such regions represent multiple copies of mobile elements or phages.

***BopGenomes*** makes a list of all unique restriction fragments; i.e. as all the genomes are considered, a fragment is added to the list only if it was not already in the list. Restriction fragments cannot be used directly to assess genome content because restriction fragments are degenerate; i.e. multiple restriction fragments can include the same homologous sequence. For instance, the appearance of a new restriction site destroys an existing restriction fragment and creates in its place two fragments whose lengths sum to the length of the original fragment. In order to deal with restriction fragment degeneracy each fragment is divided into ∼200 bp sections called “bops”. Most bops are exactly 200 bp, but bops at the end of a fragment may be less than 200 bp. If a bop is <100 bp it is joined to the previous bop, thus generating a bop of up to 300 bp. Although the use of restriction fragments may appear to be superfluous when analyzing completely sequenced genomes, it does serve to put the bops in most homologous regions into the same frame. Doing that reduces the number of unique bops and dramatically reduces computation time.

After introducing the restriction sites and creating the bops, the program lists all unique bops. As each bop is considered for addition to the list it is aligned against each of the bops already in the list by the blast2seq program (NCBI). If a bop shares ≥80% sequence identity over >50% of its length with a bop already in the list it is not added to the list. At the same time, lists of each bop in each restriction fragment are maintained. Thus, from the ordered restriction map of a genome we know which bops are present. Since we know the sequence of each bop we know the sequence information that is present in each genome.

Finally, each genome is described by a binary string in which the *i* th character indicates the presence of bop number *i* by a 1, and its absence by a 0. Note that homologous bops (those that share >80% sequence identity) are considered equivalent. Minor variation in sequence is lost to this analysis, as is the position of sequences in the genome. The binary strings that describe the presence/absence of each bop in each of the genomes are contained in the ***BopGenomes*** output file with the extension ‘.scores’.

### Clustering by Minimum Spanning Trees (MST)

Clustering by MST was carried out using the MST gold program [7]. The pairwise distances between genomes were based on the equidistant method [7].

Just as a phylogenetic tree is a graph that illustrates the relationships between individuals and their hypothetical ancestors based on identity by descent, an MST is a graph that illustrates the relationships between individuals based on identity by state. On an MST each node represents an individual and nodes are connected by edges whose lengths reflect the distance between the nodes. In this case the distance between a pair of genomes is shown as the number of differences in the state of the bop (0 or 1) divided by the number of bops.

### Predicting phenotypes and identifying predictive segments

Bops are sufficient for estimating the genetic relationships among genomes by clustering methods, but for other downstream applications (such as predicting phenotypes) it is useful to join a series of contiguous bops that have identical distributions among the genomes into “segments”. A segment is thus a series of contiguous bops that behave as a unit with respect to their presence or absence in genomes. A genome can thus also be described by a binary string that indicates the presence or absence of each of the segments. The binary strings that describe the presence/absence of each segment in each of the genomes are contained in the ***BopGenomes*** output file with the extension ‘.segScores’.

The program ***GetProbs*** uses a set of genomes whose phenotypes are known (the training set) to calculate, from the binary strings in a .segScores file, (1) the probability of a positive phenotype given that a particular segment is present, (2) the probability of a positive phenotype given that the segment is absent, and (3) ß, the probability that the presence or absence of the segment is non-random with respect to phenotype. ß is thus a measure of the degree to which the presence or absence of a segment is associated with the phenotype. That information is saved in a file with the extension ‘.pp’.

The program ***PredictPhenotypes*** uses the probabilities for each segment in a .pp file to predict the probability that a genome whose phenotype is unknown has a positive phenotype. Since segments with high ß values are more strongly associated with a particular phenotype, the segments that are used to predict phenotype are filtered to include only those segments with ß values above a chosen threshold. The probability that a genome has a positive phenotype is the sum of the probabilities of being positive given that the segment is present for all segments that are present plus the sum of the probabilities of being positive given that the segment is absent for all segments that are absent, divided by the number of segments whose ß value is above the threshold. The program lists all of the segments above the threshold ß value.

### Strains

Most of the EHEC *E. coli* and *Shigella* strains were obtained from a collection at Michigan State University that was developed by Dr. Shannon Manning. *E. coli* strains whose genomes have been sequenced were obtained from authors of the genomes sequences.

### Genomic DNA preparation

A boiling genomic prep was used to lyse cells and extract genomic DNA. Cells were suspended in 25 µl of water and were heated to 100° for 10 minutes. The samples were then cooled and centrifuged at 1000 rpm (max speed) in a benchtop centrifuge for 1 minute and 3 µls of the supernatant each sample were used as template for 10 µl PCR reactions.

### PCR Assays

PCR reactions were performed as follows:

2x Taq Mastermix (New England Biolabs^TM^ ) was used according to manufacturer instructions. Primers were used at a concentration of 500 pM. The reaction was run for 30 cycles with a denaturation temperature of 94°C, annealing temperature of 65°C, and an elongation temperature of 72°C. We used universal 16S primers as a positive control.

### Availability of programs

***BopGenomes, GetProbs,*** and ***PredictPhenotypes*** are part of the **BopGenomes Suite** that is available for Mac, Windows and Unix platforms free of charge at http://bellinghamresearchinstitute.com/software/index.html.

## Supporting Information

Figure S1 **Updated minimum spanning tree from sequenced genomes excluding plasmids.**
(DOCX)

Figure S2 **Updated minimum spanning tree from sequenced genomes including plasmids.**
(DOCX)

Table S1 Strains, accession numbers and phenotypes.
(DOCX)

Table S2 Sequences of segments that are useful as probes to detect EHEC *E. coli* strains.
(DOCX)

Table S3 Sequences of segments that are useful as probes to detect *Shigella* strains.
(DOCX)

Table S4 EHEC-specific amplification probes.
(DOCX)

Table S5 Shigella-specific amplification probes.
(DOCX)

Table S6 Amplification profiles.
(DOCX)

References S1 **Supporting information references.**
(DOCX)
