# Supplementary material for: Using Complete Genome Comparisons to Identify Sequences Whose Presence Accurately Predicts Clinically Important Phenotypes
Source: PLoS One. 2013 Jul 23;8(7):e68901. doi: 10.1371/journal.pone.0068901 (PMC3720857; doi:10.1371/journal.pone.0068901)
Supplement: Table S1 — Strains, accession numbers and phenotypes. (DOCX) [file pone.0068901.s003.docx]

| **Strain** | **ID on MSTs** | **Accession Number** | **Phenotype**  **and reference^a^** |
| --- | --- | --- | --- |
| [Escherichia coli 042](http://www.ncbi.nlm.nih.gov/bioproject/40647) | Eco042 | [FN554766](http://www.ncbi.nlm.nih.gov/nuccore/FN554766) | EAEC ([Crossman, et al. 2010](#_ENREF_4)) |
| [Escherichia coli 536](http://www.ncbi.nlm.nih.gov/bioproject/16235) | Eco536 | NC_008253.1 | ExPec ([Zhou, et al. 2010](#_ENREF_27)) |
| Escherichia coli 53638 | Eco53638 | AAKB00000000 | EIEC |
| [Escherichia coli 55989](http://www.ncbi.nlm.nih.gov/bioproject/33413) | Eco55989 | [NC_011748.1](http://www.ncbi.nlm.nih.gov/nuccore/NC_011748.1) | EAEC ([Sims and Kim 2011](#_ENREF_17)) |
| [Escherichia coli ABU 83972](http://www.ncbi.nlm.nih.gov/bioproject/38725) | EcoABU83972 | [CP001671](http://www.ncbi.nlm.nih.gov/nuccore/CP001671) | Asymptomatic bacteriuria([Zdziarski, et al. 2010](#_ENREF_26)) |
| [Escherichia coli APEC O1](http://www.ncbi.nlm.nih.gov/bioproject/16718) | EcoAPEC01 | [NC_008563.1](http://www.ncbi.nlm.nih.gov/nuccore/NC_008563.1) | ExPec ([Zhou, et al. 2010](#_ENREF_27)) |
| [Escherichia coli ATCC 8739](http://www.ncbi.nlm.nih.gov/bioproject/18083) | EcoATCC8739 | [NC_010468.1](http://www.ncbi.nlm.nih.gov/nuccore/NC_010468.1) | Commensal ([Archer, et al. 2011](#_ENREF_2)) |
| [Escherichia coli B str. REL606](http://www.ncbi.nlm.nih.gov/bioproject/18281) | EcoB_REL606 | [NC_012967.1](http://www.ncbi.nlm.nih.gov/nuccore/NC_012967.1) | Commensal ([Sims and Kim 2011](#_ENREF_17)) |
| [Escherichia coli 'BL21-Gold(DE3)pLysS AG'](http://www.ncbi.nlm.nih.gov/bioproject/30681) | EcoBL21-Gold | [NC_012947.1](http://www.ncbi.nlm.nih.gov/nuccore/NC_012947.1) | Commensal |
| [Escherichia coli BL21(DE3)](http://www.ncbi.nlm.nih.gov/bioproject/28965) | EcoBL21DE3 | [AM946981](http://www.ncbi.nlm.nih.gov/nuccore/AM946981) | Commensal ([Sims and Kim 2011](#_ENREF_17)) |
| [Escherichia coli BW2952](http://www.ncbi.nlm.nih.gov/bioproject/33775) | EcoBW2952 | [NC_012759.1](http://www.ncbi.nlm.nih.gov/nuccore/NC_012759.1) | Commensal ([Ferenci, et al. 2009](#_ENREF_5)) |
| Escherichia coli CFT073 | EcoCFT073 | [NC_004431.1](http://www.ncbi.nlm.nih.gov/nuccore/NC_004431.1) | ExPec ([Zhou, et al. 2010](#_ENREF_27)) |
| Escherichia coli str. 'clone D i2' | EcoCloneDi2 | CP002212 | ExPec ([Reeves, et al. 2011](#_ENREF_16)) |
| Escherichia coli str. 'clone D i14' | EcoCloneDi14 | CP002212 | ExPec ([Reeves, et al. 2011](#_ENREF_16)) |
| Escherichia coli DH1 | EcoDH1 | [CP001637](http://www.ncbi.nlm.nih.gov/nuccore/CP001637) | Commensal ([Suzuki, et al. 2011](#_ENREF_18)) |
| Escherichia coli E24377A | EcoE24377A | [NC_009801.1](http://www.ncbi.nlm.nih.gov/nuccore/NC_009801.1) | ETEC |
| Escherichia coli ED1a | EcoED1a | NC_011745.1 | Commensal ([Zhou, et al. 2010](#_ENREF_27)) |
| Escherichia coli ETEC H10407 | EcoETEC_H10407 | FN649414 | ETEC ([Zhou, et al. 2010](#_ENREF_27)) |
| Escherichia coli HS | EcoHS | NC_009800.1 | Commensal ([Zhou, et al. 2010](#_ENREF_27)) |
| Escherichia coli IAI1 | EcoIAI1 | NC_011741.1 | Commensal ([Zhou, et al. 2010](#_ENREF_27)) |
| Escherichia coli IAI39 | EcoIAI39 | NC_011750.1 | ExPec ([Zhou, et al. 2010](#_ENREF_27)) |
| Escherichia coli IHE3034 | EcoIHE3034 | CP001969 | ExPec ([Moriel, et al. 2010](#_ENREF_11)) |
| Escherichia coli str. K-12 substr. DH10B | EcoK12_DH10B | NC_010473.1 | Commensal ([Zhou, et al. 2010](#_ENREF_27)) |
| Escherichia coli str. K-12 substr. MG1655 | EcoK12_MG1655 | NC_000913.2 | Commensal ([Sims and Kim 2011](#_ENREF_17)) |
| Escherichia coli str. K-12 substr. W3110 | EcoK12_W3110 | AP009048 | Commensal ([Sims and Kim 2011](#_ENREF_17)) |
| Escherichia coli KO11FL | EcoKO11FL | CP002516 | Commensal ([Turner, et al. 2012](#_ENREF_20)) |
| Escherichia coli LF82 | EcoLF82 | CU651637 | AIEC ([Wine, et al. 2009](#_ENREF_22)) |
| Escherichia coli NA114 | EcoNA114 | CP002797 | ExPec ([Avasthi, et al. 2011](#_ENREF_3)) |
| Escherichia coli O103:H2 str. 12009 | EcoO103H2_12009 | NC_013353.1 | EHEC ([Sims and Kim 2011](#_ENREF_17)) |
| Escherichia coli O104:H4 2009EL-2050 | EcoO104H4_2009EL-2050 | CP003297.1 | Other pathogen |
| Escherichia coli O104:H4 2009EL-2071 | EcoO104H4_2009EL-2071 | CP003301.1 | Other pathogen |
| Escherichia coliO104:H4 2011C-3493 | EcoO104H4_2011C-3493 | CP003289.1 | Other pathogen |
| Escherichia coli O111:H- str. 11128 | EcoO111H-_11128 | NC_013364.1 | EHEC ([Sims and Kim 2011](#_ENREF_17)) |
| Escherichia coli O127:H6 str. E2348/69 | EcoO127H6_E2348_ | NC_011601.1 | EPEC ([Zhou, et al. 2010](#_ENREF_27)) |
| Escherichia coli O157:H7 str. EC4115 | EcoO157H7_EC4115 | NC_011353.1 | EHEC ([Sims and Kim 2011](#_ENREF_17)) |
| Escherichia coli O157:H7 str. EDL933 | EcoO157H7_EDL933 | NC_002655.2 | EHEC ([Sims and Kim 2011](#_ENREF_17)) |
| Escherichia coli O157:H7 str. Sakai | EcoO157H7_Sakai | NC_002695.1 | EHEC ([Sims and Kim 2011](#_ENREF_17)) |
| Escherichia coli O157:H7 str. TW14359 | EcoO157H7_TW14359 | NC_013008.1 | EHEC ([Sims and Kim 2011](#_ENREF_17)) |
| Escherichia coli O157H7 str. TW14588 | EcoO157H7_TW14588 | [CM000662.1](http://www.ncbi.nlm.nih.gov/nuccore/CM000662.1) | EHEC ([Kulasekara, et al. 2009](#_ENREF_8)) |
| Escherichia coli O26:H11 str. 11368 | EcoO26H11_str11368 | NC_013361.1 | EHEC ([Sims and Kim 2011](#_ENREF_17)) |
| Escherichia coli O55:H7 str. CB9615 | EcoO55H7_CB9615 | NC_013941.1 | EPEC ([Zhou, et al. 2010](#_ENREF_27)) |
| Escherichia coli O55:H7 str. RM12579 | EcoO55H7_RM12579 | [CP003109.1](http://www.ncbi.nlm.nih.gov/nuccore/CP003109.1) | EPEC ([Kyle, et al. 2012](#_ENREF_9)) |
| Escherichia coli O7:K1 str. CE10 | EcoO7K1_CE10 | CP003034 | ExPec |
| Escherichia coli O83:H1 str. NRG 857C | EcoO83H1_NRG857C | CP001855 | AIEC ([Allen, et al. 2008](#_ENREF_1)) |
| [Escherichia coli P12b](http://www.ncbi.nlm.nih.gov/genome/167?project_id=162061) | EcoP21B | [CP002291.1](http://www.ncbi.nlm.nih.gov/nuccore/CP002291.1) | ????? ([Liu, et al. 2012](#_ENREF_10)) |
| Escherichia coli S88 | EcoS88 | NC_011742.1 | ExPec ([Zhou, et al. 2010](#_ENREF_27)) |
| Escherichia coli SE11 | EcoSE11 | NC_011415.1 | Commensal ([Zhou, et al. 2010](#_ENREF_27)) |
| Escherichia coli SE15 | EcoSE15 | AP009378 | Commensal ([Toh, et al. 2010](#_ENREF_19)) |
| Escherichia coli SMS-3-5 | EcoSMS35 | NC_010498.1 | Commensal ([Zhou, et al. 2010](#_ENREF_27)) |
| Escherichia coli UM146 | EcoUM146 | CP002167 | AIEC ([Krause, et al. 2011](#_ENREF_7)) |
| Escherichia coli UMN026 | EcoUMN026 | NC_011751.1 | ExPec ([Zhou, et al. 2010](#_ENREF_27)) |
| Escherichia coli UMNK88 | EcoUMNK88 | CP002729 | ???????? |
| Escherichia coli UTI89 | EcoUTI89 | NC_007946.1 | ExPec ([Zhou, et al. 2010](#_ENREF_27)) |
| Escherichia coli W | EcoW | CP002185 | Commensal |
| Escherichia coli Xuzhou21 | EcoXUZhou21 | \|  \| CP001925.1 \| \| --- \| --- \| | EHEC ([Xiong, et al. 2012](#_ENREF_23)) |
| Shigella boydii CDC 3083-94 | Shibo_CDC3083-94 | NC_010658.1 | ([Pupo, et al. 2000](#_ENREF_15)) |
| Shigella boydii Sb227 | Shibo_Sb277 | NC_007613.1 | ([Yang, et al. 2005](#_ENREF_24)) |
| Shigella dysenteriae Sd197 | Shidy_Sd197 | NC_007606.1 | ([Yang, et al. 2005](#_ENREF_24)) |
| Shigella flexneri 2002017 | Shifl_2002017 | NC_004741.1 | ([Ye, et al. 2010](#_ENREF_25)) |
| Shigella flexneri 2a str. 2457T | Shifl_2a_245T | NC_004741.1 | ([Wei, et al. 2003](#_ENREF_21)) |
| Shigella flexneri 2a str. 301 | Shifl_2a_301 | NC_004337.2 | ([Jin, et al. 2002](#_ENREF_6)) |
| Shigella flexneri 5 str. 8401 | Shifl_5_8401 | NC_008258.1 | ([Nie, et al. 2006](#_ENREF_12)) |
| Shigella flexneri 5a str. M90T | Shifi_M90T | CM001474.1 | ([Onodera, et al. 2012](#_ENREF_13)) |
| Shigella sonnei Ss046 | Shiso_Ss046 | NC_008258.1 | ([Yang, et al. 2005](#_ENREF_24)) |
| Shigella sonnei 53G | Shiso_53G | HE616528.1 |  |

^a^If no reference is given the genome is a direct submission and the phenotype is taken from the GenBank file annotation
