# Supplementary material for: Using Complete Genome Comparisons to Identify Sequences Whose Presence Accurately Predicts Clinically Important Phenotypes
Source: PLoS One. 2013 Jul 23;8(7):e68901. doi: 10.1371/journal.pone.0068901 (PMC3720857; doi:10.1371/journal.pone.0068901)
Supplement: Table S2 — Sequences of segments that are useful as probes to detect EHEC E. coli strains. (DOCX) [file pone.0068901.s004.docx]

>10254

TGGCGCGGGGAGAGTAGTCGATGAATAAACAATACGAACTGGTTGTAAAATGAATATTTCTAACTGAAAAAACGTTCCATGAGGTGAGAAAAGGTCACAGGCAATCAATAACAGGACGTGATGAAAGACCCTTGCATTTGTGCGCTTTCTCTTTAGATAGCAGCAGATACTGAAAATCTGAGTTGTCGGGGAGTCAGGGATACAGCTGTGCAAGAGTTGGTCATTGTGATTCCATTGAAATCCTGTATGCCATGAAGGGCAGGATTTTATGGCTACCTGAGCTTTGGTGATAGTAAGTTGAAAATTCGCATTTTTTGCTGACATGCGTAACGAGAATCCCATAAGCAGGGAGGACTTAATTCTTCATTAACCCATGCGTTGATATTATGTTTCAGCCGTTGAAGCATCAGCGGTGTTAATGTTGTGGTAATAATATCCAGCGTTTTATGTGAGATCTTACCGTAAGGGTCTGCAAGAATGCTGCTTGTTGCTTCGTTATTATCTGCCATCAGAAGAAGTAACTCTGATTTAACGTTTTCTGTCATTAGTTGTAAAAATCTTCTGCGCAAACTTTCTTTACTGTTCATTTATATGGCTTCATTTGTTGTAATCTGCTGCGTCTCAAGGGATATGTTTATGAGAGCGACCATGAGTGTTGGATTATATACCTAACATATCAAGGGATTAGAAATCGATAAATCCCCATGAACGAAAAAATAAAATACGGCCTGTCG

>10258

TCCGGGGAATATTTGTTAGATAAAAAAGAGGAGATAATTCAATAGGGAGTTAAATTAATGCCGATAAATCTGACATCTTATTTGTGGGTACAGGGACAGAAAGTTGTCCCGGCAGTTGTGTTTTCTAATTTTACTTTAGTGGACTAAGTAAAAAGGAGTGAGATAAATGCTGCCCACTACAAATATCTCTGTAAATTCTGGAGTAATATCTTTTGAAAGTCCTGTAGATTCACCATCTAACGAGGATGTTGAAGTTGCCCTCGAAAAGTGGTGCGCTGAGGGAGAATTTAGCGAAAATCGTCATGAGGTTGCATCAAAAATACTTGATGTTATAAGTACTAATGGAGAGACTTTATCAATCAGTGAGCCAATAACAACATTACCAGACTTGCTTCCAGGTTCTCTGAAAGAACTGGTTTTGAATGGATGTACAGAGCTTAAATCAATAAACTGCTTACCCCCCAACTTATCTTCATTAAGTATGGTTGGATGCTCATCATTAGAGGTTATAAATTGCAGCATACCTGAAAATGTCATTAATTTATCTTTATGCCATTGTAGTTCTTTGAAACATATAGAAGGTTCCTTTCCTGAGGCACTCAGAAATTCCGTATATTTAAATGGCTGTAATTCATTAAATGAATCGCAATGTCAATTCCTTGCATATGATGTCAGTCAAGGCCGTGCCTGCCTGAGCAAAGCTGAGCTTACTGCTGACTTAATTTGGTTGTCGGCTAACCGAACGGGTGAAGAGTCTGCTGAAGAATTGAATTACTCTGGATGTGACTTGTCAGGTCTAAGTCTTGTAGGGCTGAATTTATCATCAGTAAATTTTTCTGGAGCAGTGCTTGATGATACAGATCTCAGGATGAGTGATTTGTCTCAGGCTGTATTGGAAAACTGTTCTTTTAAAAACTCGATTTTGAATGAATGTAATTTTTGTTATGCTAATTTATCTAATTGTATTATTAGGGCTTTGTTTGAAAACTCTAATTTCAGCAATTCCAATCTTAAAAATGCATCATTTAAAGGATCTTCATATATACAATATCCTCCAATTTTGAACGAGGCTGATTTAACAGGAGCTATTATAATTCCTGGAATGGTTTTAAGTGGTGCTATCTTAGGTGATGTAAAGGAGCTCTTTAGTGAAAAAAGTAATACCATTAATCTAGGAGGGTGTTACATAGATCTATCTGACATACAGGAAAATATATTATCTGTGTTGGATAACTATACAAAATCAAATAAATCAATTTTATTGACTATGAATACATCTGATGATAAGTATAACCATGATAAAGTAAGGGCCGCTGAAGAACTTATCAAAAAAATATCTCTTGACGAATTAGCGGCGTTCCGGCCCTATGTTAAGATGTCTTTGGCTGATTCATTTAGTATTCATCCTTATTTGAACAACGCAAATATACAGCAATGGCTCGAGCCTATATGTGATGACTTTTTTGATACTATAATGTCTTGGTTTAATAATTCAATAATGATGTATATGGAGAATGGTAGTTTATTGCAGGCAGGGATGTATTTTGAGCGACATCCAGGTGCGATGGTATCTTATAATAGTTCCTTTATACAAATTGTAATGAATGGTTCACGGCGTGATGGAATGCAGGAACGATTTAGGGAACTCTATGAAGTATATTTAAAAAATGAAAAAGTTTATCCTGTCACACAGCAGAGTGATTTTGGATTGTGCGATGGCTCTGGGAAGCCTGACTGGGATGATGATTCCGATTTGGCTTATAACTGGGTTTTGTTATCATCACAGGATGATGGTATGGCAATGATGTGTTCTTTGAGTCATATGGTTGATATGTTATCTCCTAATACATCAACTAACTGGATGTCCTTTTTTTTATATAAGGATGGAGAAGTTCAAAATACATTTGGGTATTCATTGAGCAATCTTTTTTCTGAATCATTTCCAATTTTCAGTATTCCTTATCATAAAGCTTTTTCCCAGAATTTCGTTTCTGGTATTCTGGATATACTCATTTCTGATAATGAACTCAAAGAGAGATTTATTGAGGCACTTAATTCCAATAAATCAGATTATAAAATGATTGCTGATGATCAGCAAAGGAAACTTGCCTGTGTCTGGAATCCCTTTCTTGATGGTTGGGAACTGAACGCTCAGCATGTAGATATGATTATGGGGAGCCATGTATTGAAAGATATGCCACTAAGAAAACAGGCTGAAATATTATTTTGTTTAGGGGGGGTTTTCTGTAAATACTCATCGAGTGATATGTTTGGTACAGAGTATGATTCTCCTGAGATTCTACGGAGATATGCAAATGGATTGATTGAACAAGCTTATAAAACAGATCCTCAGGTATTTGGCTCAGTTTATTATTACAATGATATTTTAGACAGGCTACAAGGAAGAAATAATGTTTTTACTTGTACCGCTGTGCTGACTGATATGCTAACGGAGCATGCAAAAGAATCTTTTCCTGAAATATTTTCATTGTATTATCCTGTTGCGTGGCGTTGATTTAGAGACCATGGATGAATATTATTGTAACACTGTCTTTTTAGGTTTGCACATGTTCAGTGGGAACATTTATTGCTCCGTTGTTATTATGTCTTGGTTTAGTGGCATGAGCCGAATGTTCTTAAAATTTACAGTGTCGAAGATGAAAGAGTACGATAGAACTCGTTGTGATTCGATTTGTCTGATATGGTGGTAATATATGAATAGGATACTGCATATATCGTAGTGCTTGAGGATGTTGATTAGGGCATATGATTTTTATATTTTTTTTGAGCAACGTTTTAAGGGAAAATTTACATATGACAACTTTGTCTGAAGAGTATCTTTGTTGTCAGTATTTTTTTTGAATGTGATAATTGATTTTGTTTTGCTGTGAATGGTCACTATAGATGAAATATGATTTTAATTACAACGAAAATTATGTTCGTTATTTCTGGGCAAATCGTGGAGAATCATATTTTATGAATTGATATTCAGATTAATATGTTTTTGTTACTATAGTAATATGGCGTAATTAATGATTGTTTTGAAAAGACTCTCAACTGGCTTTTATTCATTGAATAGTGCGTTATAAGAGGAAGTGGAATTTTAATGAAAATAACAAACTATATACTGCCAACAAGTCGTACTCATGGTTCATTCTCAACTATAAAATCATGGGACACAATGAATTATATTAAACATTTAATCAGACATACAAATGACCCTGTATTTGAAGAACAATTTTATAAAATAACACAATCTCATATTGACTTTGACAAAAGAGCTAAAGATGAAAAAAATGACACCATTAACATTTATGATAACTTTTTCTATTCATCTAATGATGATCTTGATTCTAAAATTAGAAGTATGTTAAATAATTTATATGAGAAAAGCTTAACTTTCCGAAGAATCATTAATTATTATGTGAAGGAAATAAACTTAAGTGATTATGGCTTTCTAAAATGTAAGATTTTACCAGCATATGCTTATAACTATGAGATGGAAAATGATGCCCCCCCAAAAATACTAATTCCAATTGACCATGATTTAAATTTTATTGATGCAAAATATAATGGAGAAACTTATCGGGGAAATGAAGAGTTTGCTATTAATCTTTTTCTGCAGCATATATTACATAATGACATACAAGAACAAACATCGATAGACTTATACACGAGCATAATAAATAAAGAGTTGGATAGCAATAGAAAATCATACAATAATGAAATTTTTAACAATTTCTCTTTTGATAAGTCTGTAAAGTTGAATTCATATAACTATATTGCAGATGATATAGAGCAAGTAATCGATAAAGGAAGCAAAGTTCAATTGGAGGTATATAATTTATTATCCGAAGAAAAGATATTTGAACATAAAATTATGAATAATTGGACAAGGAGCATAAAAAATATATTGACGACATATTTGTTTATGTCATCAGGAGCGGTGACAGCCAGAAATGTTCAAACCTTTTCTCCAACAATAAATAATGAGTCAAGGATTCGATTGCCGAGAGCATTGCCAGTAGGCCATCCATATCCTGAGGAACATAAGGCTTCTGGTTTCTCCCCTTTTATGATGGGGGGGCTGAGTGGTGATATTCTTCCGGAAATTTTAACGGGGAATGGACCATCTATATTTTTTAACGGAAAACATAATAACCAACATGATGGAGCTTTTGGAAAAATAATAGATTTTACCCAAAATGGAAATAAAATAAGTGCAAAAGATAAAGAAATAATAAAAAGATATATTTTTGATAAGATCAATGTTTTGATTAAAGAGTATTTCATTAGAACTGGTAAAAATTCTCATACCCCATTTGAAGTTTTTATAAAGGAGCGATTATTTAATCAATATGATATTTTTAAAACATTGGCTAGAGATATATTGGCACACCCATTAGTAATATATGATGCGGGTTACAAAAATTATCATGAGTCATTAAATGCTGCTATTGCAATAAACTCTAGACCATTACAAGAAATACATTATGGTGATGTTTTATATCATTATCATAAAAATGACATCTCTTTGGGAGTAGATACTCTTTACGGGAGGGAAAGTTTTGATATTGTACTGGATGCAATGAACGTATATAGAAAAAGCAAAAAAATGAGAGTTATTTCCAATAATGAGATGAAAAAAAGCATTAAAATATCTGAATTAGTTATCCATAATATTATAAAGAAAGGATTGACTAATTGTTTGCTTAAAAAGGATGTTCTTAATGCCAGATATGATCTTATTAGAGATATTCTTCGATATTCTTTAAATATACGACAGGGAATTAAACATGATGATGTTAATAGAATAGCGGAAAATATAATAAAAAAGTATGGTATAACTGAGGGTATGAATCCTAAACCTAGGAATGCCAGAATATCTAAAGAATTGCTTTTATTAGCTGTTGATAGACAGATTGAGTGGGCGAAAAAACATTTTATAACAAAAGATGTATTGGAAAATGTTGTGTCAAAATGTGATTTATCATCTATCTTTAATGTTAATAAAGTGCTTCAGAATACTATTCTTGAGTTTGTCCATGAAATTAATAATATATCATCTGCTCGCTGGATGTCAAAATCAGAAAAGAATAATAAACAAAAAGAGGCAATAGAAAAGTTCAAAAAAGAAGTATCCCATATGAATGGCGGGCAGCAGTTTATTTGGGGGTTTGATAAGGTTATTCAAGAAGGCTTAAGTGGATTGATTGAGTTAAGTATCGATATTAATGATAGTACAAATCATCGTGATAAGTCTTCTCTTTCTCCTGATGGGAGAGCTGTGTTACATTTTTTAGGTACAATTTGGAATATGGCGATGGGAGCTGTACCTGGTTATAATGCATTGTCTGGTGTAAGTAGCATTTTACATAGTGCTATAGTAAAAGAATCTAGCAATATCTGTGATTATATTCAGGGGGCTGTACGTATTGGAATGGACTTTGTTCCAGGCACTCGCTCTGACTTACATAGCCGTTCGCTGCAGATAAAATATGAGGCTTTGAAGCATATAGAAAAAAACATTAATGATAATATTATTTATCATCCGAGTAATAATGCTAATTTCTATTCTGTAATTGAGTCAATTGATGGTAATGACTTTATATATAACGAAAAACAATCTAAAATATTAGAAATGAAACAGGATCGTGGGGGGAATAGATATAGTGCAGTAGATCTTAACTCTTCTAAGTATGGGTATTATGAGAAAGTTGGCGGTGGTTTTTATAGATATATAGAATCCTTTAACCCCATATCTTCAGAGACACCAAATAAAATAGTCTACAAGGGGGAATCAGTAGATTTAACTAAGGAGCCAAATTCGGAATTATATTCAGGTAGGTATTCTATAAATAACAAACAGGTTAATGTTTATTTCTTTCGTGACGCTGATGGTACATTTTATAAATCAGAAGGTCTTCATGGTGGGGGAGTTATTAGATACATAGATAAACCGTATTCTCAGTTAAGAGAAGGAGATATTGGGTATGATGAGGATTTGTTGGATATATACGATGATTCTCCGGTGCTTGAAGACACGTTGCCTGCTTTATCTTCTGAAATAGTACCAACTCCAGAACATAGTATTAAACAAATTTATTCGAAAATTAAGGAGGGGCACATAGAACTGTCCGATTCAGACATCATATTGTGTCGCGGCACAACCGGTATTCAAGCTGAAAATATCGTTGAATATAAAACTGCTGGAGGGTTTCCTGATTCAAATCCAAATGTAAAAGCACCAGATGAATATATGGCACAACAGCAGGTACGTATTGGAAGAATATTGCCTGAATACACATCGGATCTTAGCGTTGCTGATCGGTTTAGTCGTGAGCATTATCTAATAGTTGTTAAAGTAAAGGCAAAATATATCACACGAGGAAGTGTTACAGAGAGTGGTTGGGTTATAGATAAGACCGCACCTGTTGAACCACTTGCGATAATTGATAGAACTTTTGGTATGAAGGAAAATATCTCAATGGTAAATGCATCGAAATAGTTTTTTTACAATCTATGTCCTGCCTCCTCTGGTAAAAACGATGCTACATCTTTGAGATGTTGCACGGCAGTACGGTGTTGACCGATATATAGTGAAGTACACATCGGTCAACGAATTACCATTGTCAGCAATATCATCCTGATAAACTCAGTACTCGTGAGCCGCTTGATGACGGGCGGAATAGCCCAAAAGGTAAACGATCCGCATGGCAGTCCGTCGGAAAGCTGCTGCTTCATACGACCGCTTAAACCGTCAGTTAGTGTCAGTATCGCTGAAGATCAGCTTCTTTTGCTGATTTACTCTGTTTTTACCTGCCCTGATGAAATACTCTTTAATCATAACATTGATTATATCGAAAATATATTTTTTGCTATCATTAAGAATATTTATATGTGGAACCGGAGTATTTTGGACGG

>10263

CAGCAAAGCCAAGGTGTCAATATTAGAAAAGAGAGTTATATCTCAAAATCAACAGACTGTTGATTTTAACTTTTGCGAATACTTTCTCCAGTCCGTCATGCATGGATTAGGATTGCTCATTTTTATACCATTTGTTGTTTTTTATAGCGATTTGCTAAAAGGGAGAAGAAAAAACGTTCTCCAAAATTAGATTGCAACTGTTTGATTTTATGGATAGAGGCATGCTGTTTACTAGACAGTAAAAAGCATGCCAAATTTGCTATTAACCTT

>10314

GAGTTAGGTGAGTATCTTTATTCAGGAAATGTCATAAAACTCAGTCAGTTATCAATTCGTTACCTACCCAATATCAACTCAATCTCATTAATAGAGACAAAACAGAGTTTGTTGCTACATCGATTATATTCAGATGAAGTACTTCAGAGAAATGGAACACTTATCCCGACACCACTACATGAAGAAAAATCAATTCCCGCTGCCAATATAAAAACAATGCTCAACAACATACCAACTTACAAAATGTTACCGCCATTCACAGAAACACAAGGTAATTGTTCTTCTGGCGCAGCCACGTTTTTACGCAAATCAGGCGCCGAAGAAAAAGATATTCTTGCATGTAGCCCCCGAAATTATGGGCTGCATCATAACATAAAAACATGGGACCCCTTGGTTAGAAATTAAGGATCCAAATATTATATATTTCATATAAAGCAAGGAAAATATTGCCTGAATAATTGTTCAGGCAATATATCCTTACATGGCACTATTATAATAAACTATTAATATAAAAACACACCAACAGAAAAAATTAAGCATCACTTGCAACAAAGGCTTCTTTTTTTGAATCAAAGTGACATTCGTCTTTTCTCATGATCA

>10393

ACTTCTCTATTCAGGGAAGCCCGTCTGATACAAAGCTTCTATTTCTCGTTAAATATTCTCCATTCTGGCGCCAAGCCCTATCTATAGCTGATTGAAAACAACTCTAAAAACGCAATAATATCAAATAGTTTATTCCTACAGCCGATCATTTTGAGTAAAATATTGACGAGAATTTCACAAAAGGTAGATTTCATGCAAACGGATAAATTAGACTATCACATAAAGTTCTTATAAGGTTATCTATATGATAAATAGCATTAATTCTTTTTTTTCCAGCATTCCTCGCAGTATATCAAGTGTTACGCGTAACAGTAGCTTCACTGCCTCACAGCACAAAAGCACACCTAACACGGTAAAAACCAGCTCACCTCTTTCTCCCAGCAACAGTCCTGCCAGTGCA

>10396

AACAGGTCGAAGTAATACCCGCACATTACCTCCAGACGCGCTGACGGTAGCAGGCATGTGTCCGTGGCAGATGTGCACGAACAGGAAGATATACAGAAACGGTCCAGGTCAGACGATCAGCGTTCAGACTCCGCTCCACACGGACACCGCGACGCAGATACGCCTCTTGAAGCATATCTGCCTCATCGATCGTACAGAACAGATAGTGAAACCAGCCATACTGAGGCGCACGAAAACGCCTCCCCTGCTTAATTTCCGGGTCGGCTTCAGAATTGTGGGATTTTATGTGTTGTGTCATCGGATTCTCCGGTGACAGCAGGTGTCAGTTGTTCAGGCTGACTGCGCGAATTGTAAGGCAATACGCCGGAATGTACAAACAGAAAACCCGTCAGTAAGACGGGCTTAACAAGCAGGGGCGGTTACTTTAATAATTTCAGTGCCTTTACATCAACTTCAACACTGCTCAGGTCTTTATCAATTTCACCCTCAATTCTTACTTTGTCTTTCGGAGAAACATTCTGCCCGGCCCATACGCTGTCATCAATATCCGTGACAATTGTCCCGCTATTGTCACGAAACTCATAACGTTCATCACCCACTTTTTTAACGATGCTCCCTTCAAGGATAACCCATGCATCATCCTTCAGTTCTTTTGCCTGCGCTACTGTTGAACGCTCTGCTTCTGGCCCCTGGAAACCACCCTGCTGTGCAAAAGCGCCAAAAGACACACCGGAAATAAGTGCTGCAATCAATACCTTTTTCATTCATAGTCCTCTTTCAGAGATGAACATTCAAACAGC

>10402

TACTCACTGTTAACCTCCTGCAACGCTACACGATACGCCTTCTTTATCCACGCCTTACTGCCATATAATTTAGTCTTCATAATAAACACACCTGCACGACTCGCCGATATCCCCGGACAGGTTAACAGCACAGCATCCACCACACGGTTATGCTTCCGAAACTCCATTACAGTACTGCTGATAACCACCTGCCCCACCGGGCCGTAATCCTGATACAGGATTTTCACGCAGACACCCTCCTGTCGAAATAAACGTAGTTATTCACTATGCGCAGCGGCATGCCTAATTTTCTGGCAATTTCCCTTCTTTGCATGCCTCTCTGATGCAGTTGCCGCGCCAGCTCAATATCACGCTGAGAATATTTTGCCGACGGGTGAAAATCACCACGTAACATCATGCTGATGCCCAGCTCCCGTGCCTTCGTTCTCACTGCCGCTTCAGTTCGTCCGATAAGTGCGCCAACGCTTTTTACCTTCCTCGTTCCCGCACACTGCCGGAGTATCATGATTTCCGCCCGGCACCACGTCTTCCACCCACTCACCGCTGCTGTTCTCTGGTGGCGGTAATATCCCGGAGAATATCCCGGCACTTGTTCAGCTCCCGCAGCGCGGCGCAGACTCGCTCCCACTTCTGAAC

>10549

TGAGTACCTGTGCGTCAGGGTAACGCAATCAGTTCAGGTTTAGCGGGGTGTCAAGTCGGTTTGGCTAACCGACCGCAGAATTAACTGTACCTTTAAAGGTCGCCCCCGATATATCTGCCTGTCAGACATATCGCCGATTCGGTATCTCACTACATAAAACTCCTTAAAAAACAATGCGATTCTGTTGGCACGAAGCTTTCAGGAAGTCAGTCCGGAGAAGCGCCAGTACGATACAGAATGAGGTTGAAAAAAACGTTCACGTCTGGTCACTTCTTCAAATGTTAAAAAACATCTCGTAAAGAGCCAGTTTATGCGGGTTCGTTGAGTAGACGTGCGAGCGTCACTTTGTCAGCTTTAACGCTGACGAAAGTGACGC
