# Supplementary material for: Using Complete Genome Comparisons to Identify Sequences Whose Presence Accurately Predicts Clinically Important Phenotypes
Source: PLoS One. 2013 Jul 23;8(7):e68901. doi: 10.1371/journal.pone.0068901 (PMC3720857; doi:10.1371/journal.pone.0068901)
Supplement: Table S3 — Sequences of segments that are useful as probes to detect Shigella strains. (DOCX) [file pone.0068901.s005.docx]

>15843T

AAGCAAATGCCTCTTAGGAATTAATCCTTTATAATCAATTAATTACAAGTGGTGTTTCGAAATCGTTCGAAATTAATTCTAAATGTCTTTCCATTCCCTCCCTCGTCCGTCACGGTATTTATCCGTCATGCTGGATGAACGATGTCCAAGGAGCGCCTGAGTGAAATCACTGCCTTTTTCTGCTGAGTGCAGGCGACCGGATAAACTTCGGATTTCATGAAATGGCGGTGGGTCACCTTCCCATTTCAATCCGGATGTGTCTCGGGCAATCCTGAACTGTGCTGCGATTGTCCTGGCTGTCTTCCCTCCGAGTAACTTTTCGTTTTTACCGTTTATTTTTTTCAAGTTATCCAGTACATCGGTAAGTGTTAAATTCAATCTGGAAATCGTGGTGGGGAGA

>15897T

AACAGAAGTCCCGGGATAATCTGGGGCTGAAAAGTGCGGCCACGATGGAAGCACAGAGCGACATTTACGACCGGACAAAAGGCCGTCTGGCGATACCCGGCGCATTCGGCTTTGGGTGTGCTTTTCTGCCTGAAGATGTTATCCGTTTTGACACTAAGAGTGATTTCCAGGCCTGGGTAAGGAATGCGCTGCCAGGTGAATATTCCGTTGCTGGCCCCTACGACATCATCATACCCGACACACGGTTTGAAGGGGTGCTCAGCATCCGGTGGACTGATGCACGCCCTGAGACAACAGAACCGCGGTACAGAGCCAAATCCCTTACTTTTTACGGCATTAACGGCCCCATTTATCACACCCGCTACTGCTACTGGCCCATATCCAGACTGACT

>15898T

GAAAATAAATATAACCACAGAAGATATTATTTACAGAATCGTGGCGAGCTCTGTCCGCAACAGATGGGGAGACCCTGACATTGGCGGGCTGATTATTGCTGCGTACCAGGGAGAAGCTGACGGTGATAAAGTCATCAGACTTGTCAGGGGGCAGTCATACAGAGGCTCACGACTGGGACCGGTGGGGATTTCAGTGCCCAGTACTCCCACCGGAACGTATATAGCATCCCCACAATTTTTCATTACGGGATGTTCAGAGCATTCATTACCGGGGTCATATTGCGCCCTGTCCGGGGTGCCGGATGCTCATGTCTCTGGCGCAATGCCCGGGCTTTTTATTCGCACATCGTGAGGAATGCACCGTGGAAATTAAAAAAACCATTAATCCCCGTTATACCGA

>15862T

TGTGTAGTTGGTTTTCCCCAACGTTTAAAAGCCTCAGATTTTGAGGAAGGGGCGGTAGCTCTTGTAATCTGTTATCAAAAGCACTTAGTTCCTGTAGTTCACATGGTAATTCTGGTAAGGCAGTAAGCTGGTTGTTGCCAACAGAAAGTTTTTGTAGAGCCGGGGGAAGATCAGGTAGCGTCTCCAGGCTATTGAGAAGGGCTGAGAGTGACTGCAAGGAAGATGGAAGAGAAGGTAAACATGTTAAAACTCTGTTAAGTGAAACATCAAGAGCGACCAGGTGAGGCGGAAGAGCCGGGAGCCTGCTCAGTCTGTTATCGCTGGCTTTAAGTACAGTTAAGGACGGAGGGAGTTCTGGCAGAGAGCGTAGCTCATTGCTAGAGATGTTAAGTTCTTGTATGTGCGGGGGCAGGTATGGGAGAGAGCGTAATCTGAGTAAACTTAAATTGAGGGCTGGCTCTTGAAAAGCCAGACATATTTTCAGTAATCGAACTGCCTGTGTTCGGTCTTCTGTTGCAGCACCTTCCTTGGCCCAGTTATCCCAGATGCGGTGATAATCCGTGATATTTTGCTCTTCTACGGATAAACGGGATATACATC

>15901T

ACGCTATCAGAACTGAGGCCAAGTATGCTTTCCGGAATTGCCGTGATCCGGTTCTGATTAAGCCAGAATATTCTTATAATATGATCATCTCTTTCTGGAAAATCTGGTATTACTTCCAAAGCATTTCTGGCTGCATCAAGTAATTCCAATGACATTGGTAACCTCGGAAGTACAGACAGGTGATTGTCACTTACATTAATATATTCTAGTGACGCAGGTAATTCAGGAAGTGCGAACAAATGGTTATCACTCACATTTATATATTCCAGCGACACTGGTAATTCAGGAAGTGCAGATAATTGATTACTGCTTGCATTCAGCTCTTTCAATGCCCTTGGTAGCTCGGGGAGCATTGATAGTTGGTTATTGCTTACATTAATTTCATCAAGATTGTCAGGCAATCGTGGTAGAGATCTGAGACCTAAACAAGATAAGTCCAACGATGTTTCACTGTTCTCCAGACATAATTGGAGCCGGGTAAAAGCAGTTTCCCTGTTTTCTCCGGAAATGCTGTTTTTAGTCCATTCAACCCATTCGGAGAGATAATTATTGTGAACATTGTCGATTGATGTAGTTCTGTAAAAAGAGACGTTTCCAGTGGATAGGGGGGGATTATTTACAGGAAGCATAATAACCTCGCAGAAGGATATCCTGATAAAATGTGATACTGGGAAATAATAAGCTAAAATAAAATATTTCAGGATTGAAATTTATTGTTTTCATTTTAAGGAAGTGATGTTGATTTAACTGTAAAAAAACGATACTATTTTCTTAGATGGTAAATGTCTCGCCTGTGCTATATTTTGTTTTTCGGTTAGTTAAATATCGTACGTTCATATTTGAACGTTCTGCCGGAATGCATTATCAATAGAGGTAAAGTCGCAACCCCAAATCGTAAAG

>15961T

CGGTTAACATATTGTCGATTGCCCATATGGAGTGAATGTTGTGAGGTAGAGGGGGGAGTTCACTAAGAAGATTCCCTATTGCGCTAATCTCTTGTAAAGAAAATGGTAAAGGTGGTAAAACTGCCAGCCCATTACATGATACATCTAATGTTTCCAGTAGTTCTGGTAAAACAGGAAGAGAACATAATTGATTATTTGAGACATGAAGCTCCTTCAAAGATATGGGGAGTGTGGGTAGTGTGATTAGTTGATTGTGGGACGCATTCAATAATTTAAGTCCTTGAGGCAAAGCAGGCAGTTCAATAAGTCTGTTATAGCTGACATTAAGCTGTGTAAGGGACGCAGGCAATGGGGAGATTAAGCTTAAATTATTTTTACTTATATTAATTGATTTAATTCCCGGGGGGATTTCAGGTAATGTTGTCAGGCCTAATTCAGACAAGTCTAGGTTCGTCTCTTGGTTTTGTAGACATGATACTAGTCGCTGAAAAGCGATGTCTCGTTGTTCTTCTTGTATGCGGTTATTTTTCCATTCAGTCCAATGGGTTAGATAACTTTCATATGCGCGGCTAGTGTCGATTGAATAAGTGGAGAACGAATTTGAAATTAATCTGTGATTGTTATTTGTCGGGAGCATAAATATCAGGGCGTATTCTTTATGTTGAGAGGTGTTGGATTCTTTTTTATCTGCTTTGCAATGTTATTGGTTCCCTTCAGCAGTAGACAGGAACTTCTGGAGGAGGCGGATATATAGTTTGTGTAAGCATATTAACTCCATATGTTATATATTGAAGAACTTCTGCTTTACACTATCCTAAACTGGATCGGTTTAATGTAAAAAAACGATACTTTTTGGGGGAGGGGGCAATTTTTCCTTAAGGTATGAAAAAAGGAGCTGAAGCTATATAATAGCTATTAATGCCACTGATATCAGTGAATCATGTATATAAAAAA

>15983T

ACAGAGACTCCCTGATCCGTGAGGACAGAGGATTGTCTTCGAGGATGATAGTGCAGGTCGGATCAAGGCTAAGTATATTTTCCGGAATGTGTGTGATGCGATTCTCGCGGCACCGGAAAAATATCTCGGTTTCCTCTGAGTGATGATTTCTTACAGGTACGGCTGGTAGGCTTTCCAGAAGATTAGTACTTACATCGAGCGCTTCCAGTGATTCAGGTAACTCAGGAAGAAATGTCAGCTGGTTATTTCTTACTGAGAGCACTTCCAGCGATGTAGGTAATTCAGGAAGCATGGTTAGCTGATTGTTATCTGCATTAATATATTCCAGCAATGCAGGCAATTCAGGAAGCATGGTTAGTTGGTTGTTATCTACATCAAGATGTTTCAGAGATGCGGGTAATTCAGGAAGTGTTGACAGGTGATTGTCACAGGCGTCAAGGTATTCCAGCGATGCTGGCAATTCTGGTAATGATATTAGGGCATTCTGAGTAATTTCCAGAACAGTGAT

>16002T

GTATCTTACTCAAGACTAATGTCTTCTGGTATAAATCCTCTGCATTGTGAATATGTAAAACATCAAAAAAAGATGAACCAAATATACGAAAAAAGCAAATATACTTACCAGGATAGAAAGCATGATATAACACTCATTCCCTTATGGCGAATATTCTGAAACACCCTCCTCATTAGGAGAGATACATTAAATACAATTTATTCACAATCTAATTCCATATCCATATGCGAGAAATTAATATGCTCAGAAATATTTCATCCTGTTTATTTCCACATATCAGCACAATTACATCCCCCAACCATTATTTGTCCGAATGGGATGATTGGGAGAAACAGGGGTTACCGGAAGAACAGCGTACTGAGGCGGTAAGAAGACTTCGTGCATGTCTTACCTCTAAGGGGCATAAACTGGACCTGCGAGCCTTGGCGCTTTCCTCGTTACCTGTACTCCCTGCTTGCATTAAAAAGCTTGATGTGAGCTGTAATAAATTAACCATCCTTACTGATCTACCTGAAAATATTAAAGAACTTATTGCAAGAGATAATTTCTTAACACATATATCTGCATTACCACATTATCTAATAACTTTGGATGTGTCCGAAAATCAATTAGAGAATCTGCCGTTATTACCAGACACCATCAAATCACTAAGCGCAGAGTATAATAGGTTATCCACACTGCCTTCATTACCCTTGAATTTAAAAAAACTTGAGGTTAGGAACAACGAACTGCAAACTCTTCCATCTCTGCCTTCTAATCTTAAGATACTTAAGGTTGCGCACAACCATCTTACTGAACTGCCCCCTTTACCTAGGAGACTGCAACTTCTTTTTGCATATAGCAATAGATTAAGCAACTTACCAAACATCCAAGAAAATATTATCATGAGAAGATTTTTTTATTTTGAAAACAACCAAATAACTACAATCCCGACAAATCTTTTTCGTTTAGATCCTCATATAACTATTGAGATTGCAAATAACCCCTTATCAGATCAAACTCTGCTATTCTTAATACAGCAAACTTCGGTTCCAAATTTTAACGGGCCTCAGTTTCGTATTTCCCTGTCAGACCAAAACAGACTGTTTTTACGCCAGATGTTGCCGCAAAATTTACATTCGCGCCATATCAGAGTCATCACTGAAGGGGGGCAGAACTTTCAGATCCCCCCTCTTCCCGAAACTGTGGCAGCCTGGTTTCCTGAAGCAGATCGTCGGGAGGTTTCTACACAATGGACTTCTTTTTCCACCGAGGAGAATTCCCGGGCATT
