# Supplementary material for: Using Complete Genome Comparisons to Identify Sequences Whose Presence Accurately Predicts Clinically Important Phenotypes
Source: PLoS One. 2013 Jul 23;8(7):e68901. doi: 10.1371/journal.pone.0068901 (PMC3720857; doi:10.1371/journal.pone.0068901)
Supplement: Table S4 — EHEC-specific amplification probes. (DOCX) [file pone.0068901.s006.docx]

| **Probe** | **Primer Sequence** | **EHEC**  **Segment ID** | **Primer Location** | **Amplicon**  **Length** |
| --- | --- | --- | --- | --- |
| EHEC 1 |  | 16002 |  | 447bp |
| FP | 5' ATGGGATGATTGGGAGAAACA 3' |  | 514…534 |  |
| RP | 5' AGATTAGAAGGCAGAGATGGAAGA 3' |  | 960…937 |  |
|  |  |  |  |  |
| EHEC 2 |  | 15862 |  | 435bp |
| FP | 5' CCGGGGGAAGATCAGGTAGC 3' |  | 560…579 |  |
| RP | 5' TATCCCGTTTATCCGTAGAAGAG 3' |  | 994…972 |  |
|  |  |  |  |  |
| EHEC 3 |  | 15961 |  |  |
| FP | 5' ATATGGAGTGAATGTTGTGAGGTA 3' |  | 225…248 | 549bp |
| RP | 5' CTAGCCGCGCATATGAAAGTTA 3' |  | 773…752 |  |
|  |  |  |  |  |
| EHEC 4 |  | 15901 |  |  |
| FP | 5' CTCGGAAGTACAGACAGGTGATTG 3' |  | 263…286 | 483bp |
| RP | 5' CTTCTGCGAGGTTATTATGCTTCC 3' |  | 745…722 |  |
|  |  |  |  |  |
| EHEC 5 | 5' CGGGGGCGGGCCTCTGTTT 3' | 15861 | 104…122 | 551bp |
| FP | 5' TAATGATACCGGCGCTCTGCTCTC 3' |  | 654…631 |  |
| RP |  |  |  |  |
|  |  |  |  |  |
| EHEC 6 |  | 15897 |  |  |
| FP | 5' ATTCGGCTTTGGGTGTGCTTTTCT 3' |  | 104…127 | 207bp |
| RP | 5' CTGTACCGCGGTTCTGTTGTCTCA 3' |  | 310…287 |  |
|  |  |  |  |  |
| EHEC 7 |  | 15843 |  |  |
| FP | 5' GGAGCGCCTGAGTGAAAT 3' |  | 150…167 | 158bp |
| RP | 5' AGGGAAGACAGCCAGGACAATC 3' |  | 307…286 |  |
|  |  |  |  |  |
| EHEC 8 |  | 15983T |  |  |
| FP | 5' GGATTGTCTTCGAGGATGATAGTG 3' |  | 30…53 | 419bp |
| RP | 5' CTGGAATACCTTGACGCCTGTGAC 3' |  | 448…425 |  |
| EHEC 1 |  |  |  |  |
