# Supplementary material for: Using Complete Genome Comparisons to Identify Sequences Whose Presence Accurately Predicts Clinically Important Phenotypes
Source: PLoS One. 2013 Jul 23;8(7):e68901. doi: 10.1371/journal.pone.0068901 (PMC3720857; doi:10.1371/journal.pone.0068901)
Supplement: Table S5 — Shigella-specific amplification probes. (DOCX) [file pone.0068901.s007.docx]

| **Probe** | **Primer Sequence** | **Shigella**  **Segment ID** | **Primer Location** | **Amplicon**  **Length** |
| --- | --- | --- | --- | --- |
| Shi 1 |  | 16002T |  | 447bp |
| FP | 5' ATGGGATGATTGGGAGAAACA 3' |  | 314…334 |  |
| RP | 5' AGATTAGAAGGCAGAGATGGAAGA 3' |  | 760…737 |  |
|  |  |  |  |  |
| Shi 2 |  | 15862T |  | 435bp |
| FP | 5' CCGGGGGAAGATCAGGTAGC 3' |  | 160…179 |  |
| RP | 5' TATCCCGTTTATCCGTAGAAGAG 3' |  | 594…572 |  |
|  |  |  |  |  |
| Shi 3 |  | 15961T |  | 549bp |
| FP | 5' ATATGGAGTGAATGTTGTGAGGTA 3' |  | 25…48 |  |
| RP | 5' CTAGCCGCGCATATGAAAGTTA 3' |  | 573…552 |  |
|  |  |  |  |  |
| Shi 4 |  | 15901T |  | 483bp |
| FP | 5' CTCGGAAGTACAGACAGGTGATTG 3' |  | 163…186 |  |
| RP | 5' CTTCTGCGAGGTTATTATGCTTCC 3' |  | 645…622 |  |
|  |  |  |  |  |
| Shi 5 |  | 15898T |  | 338 |
| FP | 5' CCGCAACAGATGGGGAGAC 3' |  | 55…73 |  |
| RP | 5' ACGGGGATTAATGGTTTTT 3' |  |  |  |
|  |  |  |  |  |
| Shi 6 |  | 15897T |  | 207bp |
| FP | 5' ATTCGGCTTTGGGTGTGCTTTTCT 3' |  | 104…127 |  |
| RP | 5' CTGTACCGCGGTTCTGTTGTCTCA 3' |  | 310…287 |  |
|  |  |  |  |  |
| Shi 7 |  | 15843T |  | 158bp |
| FP | 5' GGAGCGCCTGAGTGAAAT 3' |  | 150…167 |  |
| RP | 5' AGGGAAGACAGCCAGGACAATC 3' |  | 307…286 |  |
|  |  |  |  |  |
| Shi 8 |  | 15983T |  | 419 bp |
| FP | 5' GGATTGTCTTCGAGGATGATAGTG 3' |  | 30…53 |  |
| RP | 5' CTGGAATACCTTGACGCCTGTGAC 3' |  | 448…425 |  |
