# Supplementary material for: Using Complete Genome Comparisons to Identify Sequences Whose Presence Accurately Predicts Clinically Important Phenotypes
Source: PLoS One. 2013 Jul 23;8(7):e68901. doi: 10.1371/journal.pone.0068901 (PMC3720857; doi:10.1371/journal.pone.0068901)
Supplement: Table S6 — Amplification profiles. (DOCX) [file pone.0068901.s008.docx]

| **EHEC-specific probes** | | |
| --- | --- | --- |
| **Strain^a^** | **Amplification Profile^b^** | **Probability**  **EHEC** |
| *Escherichia coli* O55:H7 RM12579 | 1000 | 0.98 |
| ***Escherichia coli* E32511** | 1110 | >0.9999 |
| ***Escherichia coli* G5101** | 1110 | >0.9999 |
| ***Escherichia coli* 5905** | 1110 | >0.9999 |
| ***Escherichia coli* DEC8C** | 1011 | >0.9999 |
| ***Escherichia coli* DEC10B** | 1011 | >0.9999 |
| ***Escherichia coli* DEC10C** | 1011 | >0.9999 |
| ***Escherichia coli* DEC9F** | 1100 | >0.9998 |
| ***Escherichia coli* VP30** | 1011 | >0.9999 |
| ***Escherichia coli* RDEC-1** | 1000 | 0.98 |
| ***Escherichia coli* MT#10** | 0111 | >0.9999 |
| ***Escherichia coli* M103-19** | 1011 | >0.9999 |
| ***Escherichia coli* MI01-88** | 1011 | >0.9999 |
| ***Escherichia coli* MI05-14** | 1011 | >0.9999 |
| ***Escherichia coli* DA-21** | 1011 | >0.9999 |
| ***Escherichia coli* RD8** | 0000 | 0 |
| ***Escherichia coli* DA-5** | 0011 | 0.9999 |
| ***Escherichia coli* IH 16** | 0111 | >0.9999 |
| ***Shigella*-specific probes** | | |
| **Strain^a^** | **Amplification Profile** | **Probability**  ***Shigella*** |
| *Shigella sp.* 2770-51 | 11011111 | >0.9999 |
| *Shigella sp.* K-147 | 10001001 | >0.9999 |
| *Shigella sp.* 3554-77 | 00011001 | 0.9998 |
| *Shigella flexneri*  2457T | 11111110 | >0.9999 |
| *Escherichia coli* 53638 | 11111101 | >0.9999 |
| *Escherichia coli* H10407 | 00010000 | 0.893 |
| ***Escherichia coli* EC4115** | 00010000 | 0.893 |
| *Sodalis glossinidius* str 'morsitans DNA' | 00000010 | 0.96 |
| *Citrobacter rodentium* ICC168 | 00001000 | 0.963 |

^a^Boldface strains are EHEC

^b^EHEC amplification profiles based on probes EHEC1, EHEC4, EHEC5, EHEC6
