# Supplementary material for: Using Complete Genome Comparisons to Identify Sequences Whose Presence Accurately Predicts Clinically Important Phenotypes
Source: PLoS One. 2013 Jul 23;8(7):e68901. doi: 10.1371/journal.pone.0068901 (PMC3720857; doi:10.1371/journal.pone.0068901)
Supplement: References S1 — Supporting information references. (DOCX) [file pone.0068901.s009.docx]

Reference

Supplementary Material References

Allen CA, Niesel DW, Torres AG 2008. The effects of low-shear stress on Adherent-invasive Escherichia coli. Environ Microbiol 10: 1512-1525

Archer CT, Kim JF, Jeong H, Park JH, Vickers CE, Lee SY, Nielsen LK 2011. The genome sequence of E. coli W (ATCC 9637): comparative genome analysis and an improved genome-scale reconstruction of E. coli. BMC Genomics 12: 9

Avasthi TS, Kumar N, Baddam R, Hussain A, Nandanwar N, Jadhav S, Ahmed N 2011. Genome of multidrug-resistant uropathogenic Escherichia coli strain NA114 from India. J Bacteriol 193: 4272-4273

Crossman LC, Chaudhuri RR, Beatson SA, et al. 2010. A commensal gone bad: complete genome sequence of the prototypical enterotoxigenic Escherichia coli strain H10407. J Bacteriol 192: 5822-5831

Ferenci T, Zhou Z, Betteridge T, Ren Y, Liu Y, Feng L, Reeves PR, Wang L 2009. Genomic sequencing reveals regulatory mutations and recombinational events in the widely used MC4100 lineage of Escherichia coli K-12. J Bacteriol 191: 4025-4029

Jin Q, Yuan Z, Xu J, et al. 2002. Genome sequence of Shigella flexneri 2a: insights into pathogenicity through comparison with genomes of Escherichia coli K12 and O157. Nucleic Acids Res 30: 4432-4441

Krause DO, Little AC, Dowd SE, Bernstein CN 2011. Complete genome sequence of adherent invasive Escherichia coli UM146 isolated from Ileal Crohn's disease biopsy tissue. J Bacteriol 193: 583

Kulasekara BR, Jacobs M, Zhou Y, et al. 2009. Analysis of the genome of the Escherichia coli O157:H7 2006 spinach-associated outbreak isolate indicates candidate genes that may enhance virulence. Infect Immun 77: 3713-3721

Kyle JL, Cummings CA, Parker CT, et al. 2012. Escherichia coli serotype O55:H7 diversity supports parallel acquisition of bacteriophage at Shiga toxin phage insertion sites during evolution of the O157:H7 lineage. J Bacteriol 194: 1885-1896

Liu B, Hu B, Zhou Z, Guo D, Guo X, Ding P, Feng L, Wang L 2012. A novel non-homologous recombination-mediated mechanism for Escherichia coli unilateral flagellar phase variation. Nucleic Acids Res 40: 4530-4538

Moriel DG, Bertoldi I, Spagnuolo A, et al. 2010. Identification of protective and broadly conserved vaccine antigens from the genome of extraintestinal pathogenic Escherichia coli. Proc Natl Acad Sci U S A 107: 9072-9077

Nie H, Yang F, Zhang X, et al. 2006. Complete genome sequence of Shigella flexneri 5b and comparison with Shigella flexneri 2a. BMC Genomics 7: 173

Onodera NT, Ryu J, Durbic T, Nislow C, Archibald JM, Rohde JR 2012. Genome sequence of Shigella flexneri serotype 5a strain M90T Sm. J Bacteriol 194: 3022

Petty NK, Bulgin R, Crepin VF, et al. 2010. The Citrobacter rodentium genome sequence reveals convergent evolution with human pathogenic Escherichia coli. J Bacteriol 192: 525-538

Pupo GM, Lan R, Reeves PR 2000. Multiple independent origins of Shigella clones of Escherichia coli and convergent evolution of many of their characteristics. Proc Natl Acad Sci U S A 97: 10567-10572

Reeves PR, Liu B, Zhou Z, et al. 2011. Rates of mutation and host transmission for an Escherichia coli clone over 3 years. PLoS One 6: e26907

Sims GE, Kim SH 2011. Whole-genome phylogeny of Escherichia coli/Shigella group by feature frequency profiles (FFPs). Proceedings of the National Academy of Sciences of the United States of America 108: 8329-8334

Suzuki S, Ono N, Furusawa C, Ying BW, Yomo T 2011. Comparison of sequence reads obtained from three next-generation sequencing platforms. PLoS One 6: e19534

Toh H, Oshima K, Toyoda A, et al. 2010. Complete genome sequence of the wild-type commensal Escherichia coli strain SE15, belonging to phylogenetic group B2. J Bacteriol 192: 1165-1166

Turner PC, Yomano LP, Jarboe LR, York SW, Baggett CL, Moritz BE, Zentz EB, Shanmugam KT, Ingram LO 2012. Optical mapping and sequencing of the Escherichia coli KO11 genome reveal extensive chromosomal rearrangements, and multiple tandem copies of the Zymomonas mobilis pdc and adhB genes. J Ind Microbiol Biotechnol 39: 629-639

Wei J, Goldberg MB, Burland V, et al. 2003. Complete genome sequence and comparative genomics of Shigella flexneri serotype 2a strain 2457T. Infect Immun 71: 2775-2786

Wine E, Ossa JC, Gray-Owen SD, Sherman PM 2009. Adherent-invasive Escherichia coli, strain LF82 disrupts apical junctional complexes in polarized epithelia. BMC Microbiol 9: 180

Xiong Y, Wang P, Lan R, et al. 2012. A novel Escherichia coli O157:H7 clone causing a major hemolytic uremic syndrome outbreak in China. PLoS One 7: e36144

Yang F, Yang J, Zhang X, et al. 2005. Genome dynamics and diversity of Shigella species, the etiologic agents of bacillary dysentery. Nucleic Acids Res 33: 6445-6458

Ye C, Lan R, Xia S, et al. 2010. Emergence of a new multidrug-resistant serotype X variant in an epidemic clone of Shigella flexneri. J Clin Microbiol 48: 419-426

Zdziarski J, Brzuszkiewicz E, Wullt B, et al. 2010. Host imprints on bacterial genomes--rapid, divergent evolution in individual patients. PLoS Pathog 6: e1001078

Zhou Z, Li X, Liu B, et al. 2010. Derivation of Escherichia coli O157:H7 from its O55:H7 precursor. PLoS One 5: e8700
